# Mutant NPM1 modulates PDCD4 ubiquitination degradation and facilitates leukemogenesis

**DOI:** 10.1016/j.isci.2025.113776

**Published:** 2025-10-15

**Authors:** Chuangxuan Liang, Jing Ke, Zhenyu Zhang, Huarong Guo, Hongxin Shang, Danwen Liu, Shan Li, Fuyun Wu

**Affiliations:** 1 School of Basic Medical Sciences, Hubei University of Medicine, Shiyan, China; 2 Institute of Geriatric Medicine, Sinopharm Dongfeng General Hospital, Shiyan, China

**Keywords:** Molecular biology, Cell biology

## Abstract

PDCD4 is a nuclear-cytoplasmic shuttling protein. It functions as a protein translation inhibitor and regulates cancer development. Here, we show that PDCD4 interacts with NPM1. NPM1 mutation results in cytoplasmic localization of mutated protein, NPMc+, which plays critical roles in leukemogenesis. We demonstrate that NPMc+ induces abnormal localization of PDCD4 in the cytoplasm and accelerates its ubiquitination degradation. Additionally, we uncover the function of PDCD4 in regulating histone deacetylation and gene transcription in the nucleus. These results imply that NPMc+ may initiate leukemia at both the transcriptional and translational levels by modulating the mislocalization and degradation of PDCD4. Finally, we show that the use of PDCD4-derived peptides to block the interaction between NPMc+ and PDCD4 exhibits a promising therapeutic effect in NPM1-mutated acute myeloid leukemia (AML) mice. Our findings suggest that the NPMc+/PDCD4 complex could be a potential therapeutic target for this subtype of AML.

## Highlights

• NPMc+ regulates PDCD4 ubiquitination-mediated degradation to promote leukemogenesis
• NPMc+ may induce leukemia at transcriptional and translational levels via PDCD4
• Block PDCD4-NPMc+ interaction with peptide: promising therapeutic for NPM1-mut AML

## Graphical abstract

**Figure:**
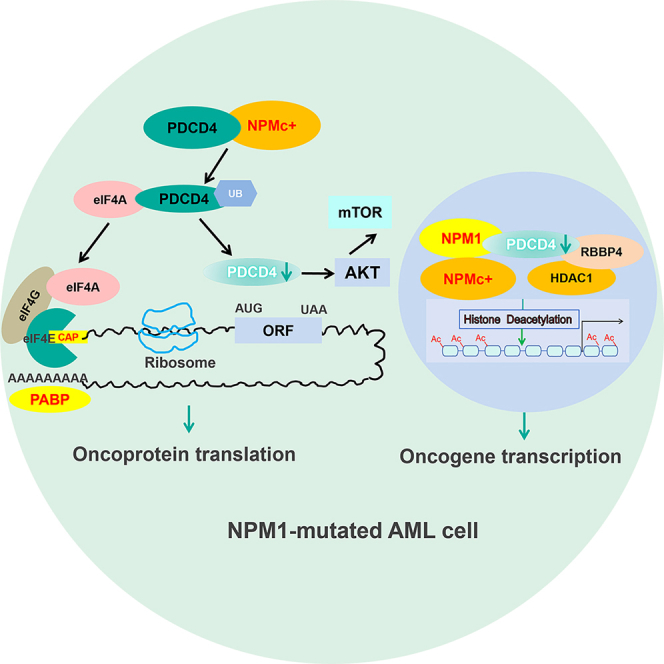

## Introduction

PDCD4, a multifunctional nuclear-cytoplasmic shuttling protein, has been intricately linked to the development of diverse cancer types. Although the loss of PDCD4 expression has been associated with tumorigenesis and tumor progression in solid tumors.^1^^,^^2^^,^^3^^,^^4^ Its role in hematopoietic malignancies, especially acute myeloid leukemia (AML), remains poorly understood. Previous studies have shown that microRNA-21, an important regulator of PDCD4, is involved in maintaining hematopoietic stem cell homeostasis.^5^^,^^6^ MicroRNA-21 is overexpressed in NPM1-mutant AML and promoted the proliferation of AML cells.^7^^,^^8^ In AML, higher PDCD4 expression correlates with a more favorable prognosis following chemotherapy,^9^ which indicated that PDCD4 play essential roles in the pathogenesis of AML.

PDCD4 functions as a tumor-suppressor gene through regulation of cell growth, apoptosis, tumor invasion, and metastasis.^10^^,^^11^^,^^12^ Its main biological function appears to involve the protein translation via binding to the initiation factor eIF4A.^13^^,^^14^ The loss of PDCD4 may lead to dysregulated gene expression and function in hematopoietic stem/progenitor cells. Moreover, PDCD4 regulates multiple oncogenic signaling pathways, such as PI3K/AKT pathway, hedgehog pathway, and NF-κB signal pathway, which are aberrant activation in AML cells.^15^^,^^16^^,^^17^^,^^18^ Although the role of PDCD4-mediated translation inhibition has been extensively studied in tumor development, its nuclear function remains poorly understood. There is increasing evidence suggest that PDCD4 is involved in transcriptional regulation. PDCD4 interacts with many kinds of transcription factors. For example, TWIST1, a transcription factor-encoding oncogene, is widely recognized for its role in AML.^19^ It has been reported that PDCD4 inhibits NF-κB-dependent transcription by directly interacting with the p65.^20^ Knockdown of PDCD4 dramatically increases AP-1-dependent transcription.^21^

Here, we identified Nucleophosmin 1 (NPM1) as a PDCD4-interacting protein. NPM1, a widely expressed phosphoprotein, is primarily located in the nucleolus and shuttles between the cytoplasm and the nucleus.^22^ It performs crucial functions, including ribosome biogenesis, centrosome replication, cell-cycle regulation, and maintenance of genome stability.^23^ The NPM1 protein consists of three important structural domains, an N-terminal oligomerization domain, a central domain, and a C-terminal nucleic acid-binding domain.^24^ The C-terminal domain contains the nucleolar localization signal (NoLS), which is responsible for its nucleolar localization.^25^ NPM1 is highly expressed in various solid tumors and is considered an important target for tumor therapy.^26^^,^^27^ NPM1 is also one of the most common genetic mutations in AML and about 30% of AML patients with mutations in the 12th exon of NPM1, which leads to a frameshift mutation at the C-terminus, producing an additional leucine and valine-rich nuclear export signal (NES) and resulting in the cytoplasmic localization of mutant NPM1 protein, NPMc+.^28^^,^^29^ The aberrant localization of NPMc+ can impact the subcellular localization and functional activity of its interacting proteins.^30^ NPMc+ triggers the cytoplasmic mislocalization of multiple nuclear proteins involved in apoptosis, DNA repair, and cellular differentiation, such as ARF, HEXIM1, FBW7, and caspases 6 and 8.^31^^,^^32^ Recent investigations demonstrate that NPMc+ maintains the leukemic state through HOX gene overexpression.^33^ Furthermore, NPMc+ directly associates with active chromatin regions and usurps the transcriptional regulation of AML-driving genes.^34^

In this study, we examined whether NPMc+ induces leukemia by interacting with PDCD4 and affecting its cytoplasmic and nuclear functions. Our results reveal a pathogenic mechanism in NPM1-mutated AML, suggesting that the NPMc+/PDCD4 complex could be a potential therapeutic target for this subtype of AML.

## Results

### PDCD4 is associated with the progression of AML

**Figure 1:**
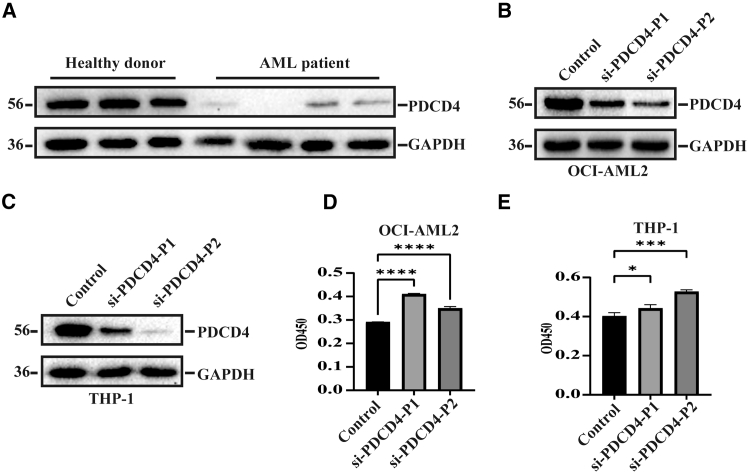
PDCD4 is associated with the progression of AML (A) Western blotting analysis to detect the expression level of PDCD4 in clinical AML samples. (B) Western blotting analysis detection of the siRNA knockdown efficiency of PDCD4 in the OCI-AML2 cells. (C) As in B, but in the THP-1 cells. (D) CCK-8 assay to assess the effect of PDCD4 knockdown on the proliferation of OCI-AML2 cells. (E) As in B, but CCK-8 assay was performed in the THP-1 cells. All data are presented as mean ± SD of three independent experiments. *p* value is calculated using one-way ANOVA. ∗*p* < 0.05; ∗∗∗*p* < 0.001 and ∗∗∗∗*p* < 0.0001.

To explore the relationship between PDCD4 and AML, peripheral blood mononuclear cells (PBMCs) were isolated from newly diagnosed AML patients and healthy donors. Total proteins extracted from these cells were then analyzed via western blotting to detect the expression level of PDCD4. As shown in Figure 1A, a notable reduction in PDCD4 protein expression was observed in AML samples (Figure 1A). To determine whether the loss of PDCD4 contributed to the progression of AML, the expression of PDCD4 was knocked down by siRNA in two AML cell lines, OCI-AML2 and THP-1 cells (Figures 1B and 1C). Subsequently, cell viability was assessed using the CCK-8 assay. As expected, the decreased expression of PDCD4 significantly promoted the proliferation of AML cells (Figures 1D and 1E). Flow cytometry was utilized to assess the cell cycle and detect cellular apoptosis. Knockdown of PDCD4 was observed to reduce apoptosis and accelerate cell cycle progression in AML cells (Figure S1). These findings indicated that PDCD4 was implicated in the development of AML.

### Identification of NPM1 as the interaction partner of PDCD4

**Figure 2:**
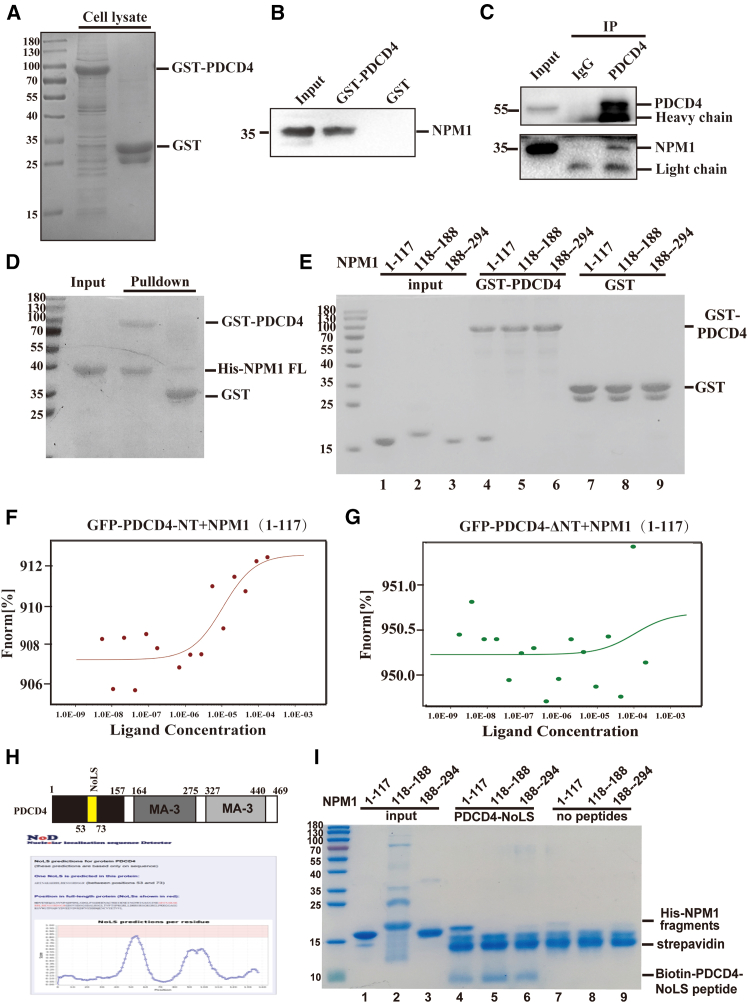
Identification of NPM1 as the interaction partner of PDCD4 (A) GST Pull-down assay to determine interaction proteins of PDCD4. GST-PDCD4 and GST proteins were immobilized on glutathione Sepharose beads and incubated with the cell lysate. The pull-down proteins were subjected to SDS-PAGE and analyzed by mass spectrometry. (B) The pull-down samples were analyzed by western blotting using anti-NPM1 antibody. (C) Validation of interactions between PDCD4 and NPM1 by co-immunoprecipitation assays. IgG was used as negative control. (D) As in A, GST pull-down assay to detect the interaction between PDCD4 and the purified NPM1 proteins. (E) As in D, GST pull-down assay to detect the interaction between PDCD4 and NPM1 fragments proteins. (F) MST analysis of interaction between NPM1-NT (1–117) and GFP-PDCD4-NT. (G) As in F, but with GFP-PDCD4-ΔNT. (H) Identification of the nucleolar localization signal (NoLS) in PDCD4 proteins. (I) Peptide-based pull-down assay to detect the binding ability of PDCD4-NoLS motif and NPM1 fragments.

To uncover the mechanism by which PDCD4 influences the progression of AML, we performed the GST pull-down to identify proteins that interact with PDCD4. GST and GST-tagged PDCD4 proteins were expressed and purified in *E. coli* and used to pull-down potential interacting proteins from the cell lysate. The pull-down samples were separated by SDS-PAGE and subsequently analyzed via mass spectrometry (Figure 2A). Through this approach, we identified NPM1 as a binding partner of PDCD4. The interaction was further verified by western blotting using an anti-NPM1 antibody (Figure 2B). To confirm the interaction between endogenous PDCD4 and NPM1 *in vivo*, co-immunoprecipitation was carried out in whole cell lysates with protein G Dynabeads, PDCD4 antibody and IgG. The immunoprecipitates were analyzed by western blotting with PDCD4 and NPM1 antibodies. As shown in Figure 2C, NPM1 was co-immunoprecipitated with PDCD4 by an anti-PDCD4 antibody, while the control IgG failed to do so (Figure 2C). Next, we explored the binding domain of PDCD4 and NPM1. His-tagged NPM1 full-length and truncated proteins were expressed and purified in *E. coli*. These were then incubated with the GST-PDCD4 immobilized on glutathione-agarose beads. GST pull-down assay demonstrated the direct interaction between PDCD4 and NPM1 (Figure 2D), and it was found that the N-terminal of NPM1 (NPM1—NT, aa 1–117) was the binding domain for PDCD4 (Figure 2E, lane 4). Next, we sought to determine the domain of PDCD4 responsible for interacting with NPM1-NT. However, the N terminal of PDCD4 (aa 1–157) was difficult to purify. Thus, we utilized the microscale thermophoresis (MST) assay to measure the binding affinity between NPM1-NT (as the ligand) and GFP-PDCD4-NT as well as GFP-PDCD4-ΔNT proteins (as the fluorescent targets), which were overexpressed in HEK293T cells. The MST results indicated that PDCD4-NT had a strong binding capacity with NPM1-NT (Figure 2F), while no binding was observed between PDCD4-ΔNT and NPM1-NT (Figure 2G). Upon analyzing the amino acid sequence of PDCD4-NT, we noticed a positively charged region within the NLS motif (aa 58–64). Since it has been previously reported that NPM1 interacts with nearly all proteins containing a nucleolar localization signal (NoLS), we conducted a bioinformatic analysis of PDCD4 using the nucleolar localization signal detector (NoD). This analysis identified a NoLS spanning residues 53–73, which overlapped with the NLS motif (Figure 2H). To test whether PDCD4-NoLS peptides bind to NPM1, synthesized biotinylated PDCD4-NoLS peptides were immobilized on streptavidin beads and incubated with NPM1 fragments proteins. As expected, PDCD4-NoLS peptides interacted with NPM1-NT domain (Figure 2I, lane 4).

### NPMc+ interferes with the cellular localization and expression of PDCD4

**Figure 3:**
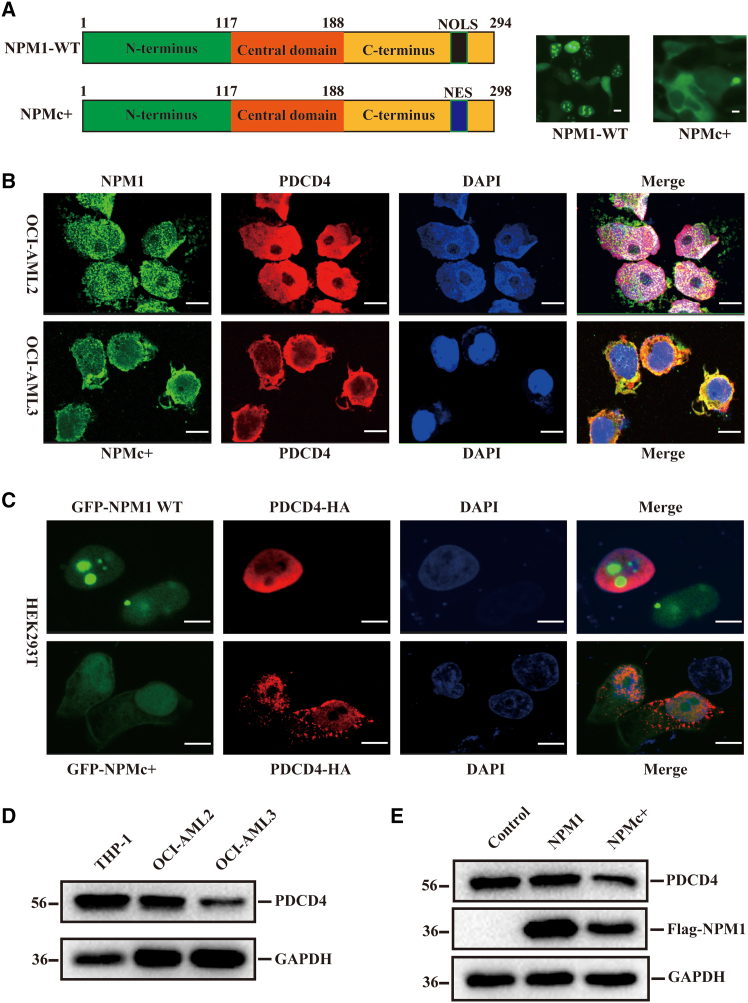
NPMc+ interferes with the cellular localization and expression of PDCD4 (A) Schematic of NPM1 domain structure and NPM1 mutations in AML (left) and subcellular localization of NPM1 and NPMc+ (right). Scale bar: 5 μm. (B) Co-localization of NPM1 (green) and PDCD4 (red) in OCI-AML2 and OCI-AML3 cells. Cell nuclei were counterstained with DAPI. Scale bar: 5 μm. (C) Immunofluorescence assay to detect the subcellular localization of PDCD4 in HEK293T cells overexpressing with GFP-NPM1 or GFP-NPMc+. Scale bar: 5 μm. (D) Western blotting analysis to detect the expression level of PDCD4 in AML cells. (E) Western blotting analysis to detect the expression level of PDCD4 in the HEK293T cells overexpressing GFP-NPM1 or GFP-NPMc+.

NPM1 is the gene most frequently mutated in AML. A 4-bp frameshift insertion in exon 12 causes the loss of the NoLS and generates a nuclear export signal (NES) at the C-terminal of NPM1. Mutations in the NPM1 gene result in the abnormal cytoplasmic localization of the mutant protein, NPMc+ (Figure 3A). We hypothesized that an abnormal interaction might occur between NPMc+ and PDCD4 in the cytoplasm of NPM1-mutated AML cells. To test this hypothesis, we observed the localization of PDCD4 and NPM1 by immunofluorescence in the NPM1 wild-type AML cell line, OCI-AML2 and the NPM1-mutated cell line, OCI-AML3. As anticipated, in OCI-AML2 cells, NPM1 and PDCD4 co-localized in the nucleus. However, in OCI-AML3 cells, NPMc+ was present in the cytoplasm, where it extensively co-localized with PDCD4 (Figure 3B). We further confirmed the location of PDCD4 in HEK293T cells overexpressing GFP-NPMc+ through immunofluorescence. As expected, PDCD4 was abnormally located in the cytoplasm (Figure 3C). Subsequently, we detected the expression level of PDCD4 in THP-1, OCI-AML2, and OCI-AML3 cells via western blotting. The results indicated lower PDCD4 protein expression in OCI-AML3 cells (Figure 3D). Similarly, in HEK293T cells, the expression level of PDCD4 was significantly reduced in cells overexpressing GFP-NPMc+ compared to those overexpressing GFP-NPM1-WT (Figure 3E). These findings suggest that the interaction between NPMc+ and PDCD4 not only alters the localization of PDCD4 from the nucleus to the cytoplasm but also decreases the expression of PDCD4.

### NPMc+ induced the ubiquitination degradation of PDCD4

**Figure 4:**
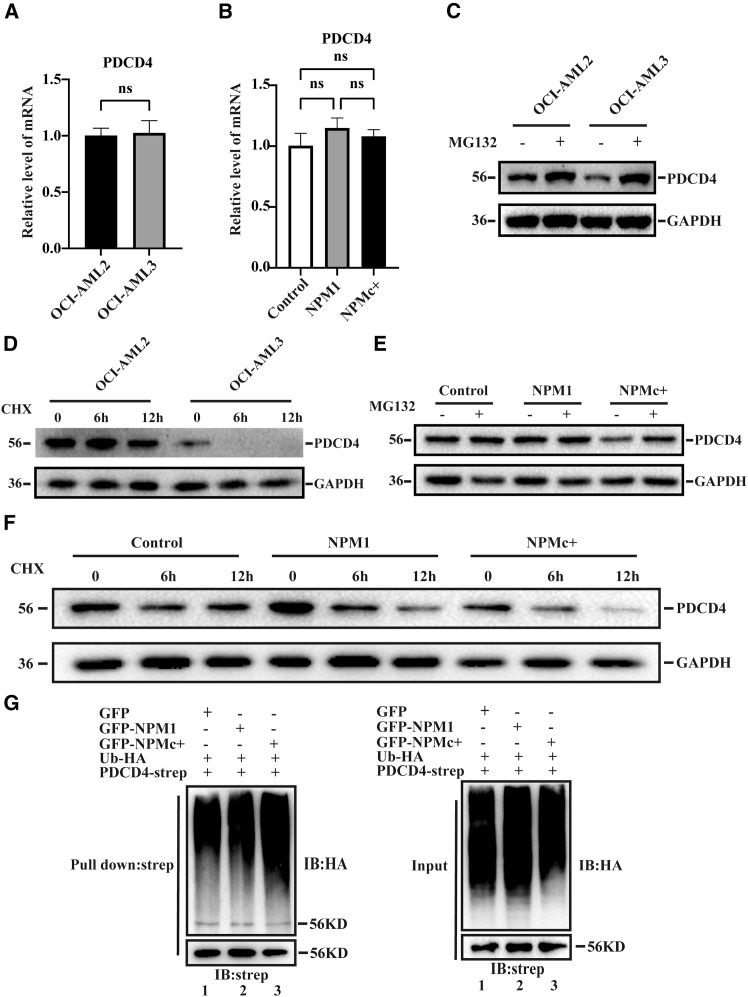
NPMc+ induced the ubiquitination degradation of PDCD4 (A) RT-qPCR analysis of mRNA levels of PDCD4 in OCI-AML2 and OCI-AML3 cells. (B) RT-qPCR analysis of mRNA levels of PDCD4 in the HEK293T cells overexpressing GFP-NPM1 or GFP-NPMc+. (C) Western blotting analysis of the expression level of PDCD4 in AML cells treated with MG132. (D) Western blotting analysis of the expression level of PDCD4 in AML cells treated with CHX for the indicated time. (E) Western blotting analysis showing the expression level of PDCD4 in HEK293T cells overexpressing GFP-NPM1 or GFP-NPMc+ treated with MG132. (F) As in E, but the HEK293T cells treated with CHX for the indicated time. (G) Western blot analysis of the levels of ubiquitin-conjugated PDCD4 in HEK293T cells overexpressing with GFP-NPM1 or GFP-NPMc+. All data are presented as mean ± SD of three independent experiments. *p* value is calculated using the unpaired two-tailed *t* test (two groups) and one-way ANOVA (more than two groups). ns, not significantly different.

To investigate the mechanism by which NPMc+ leads to a decrease in PDCD4 expression, the transcription of PDCD4 was analyzed by RT-qPCR in OCI-AML2 and OCI-AML3 cells. No difference in the mRNA level of PDCD4 was observed between the two AML cell lines (Figure 4A). Similarly, in HEK293T cells overexpressing GFP-NPM1 or GFP-NPMc+, there was no significant difference in the PDCD4 mRNA level (Figure 4B). These findings indicate that NPMc+ does not impact the transcription of PDCD4. Next, we investigated whether NPMc+ mediated the ubiquitination degradation of PDCD4. After treating the cells with proteasome inhibitor MG132, the PDCD4 protein expression in AML cells increased significantly (Figure 4C). Conversely, when treated with cycloheximide (CHX, a protein synthesis inhibitor), the PDCD4 protein degraded rapidly in OCI-AML3 cells (Figure 4D). As anticipated, the reduction of PDCD4 mediated by NPMc+ was alleviated after treatment with MG132 (Figure 4E), whereas NPMc+ enhanced the degradation of PDCD4 following CHX treatment (Figure 4F). Next, we examined whether NPMc+ is involved in the ubiquitination degradation of PDCD4. Strep-tagged PDCD4, HA-Ub and either GFP-NPM1 or GFP-NPMc+ were co-expressed in HEK293T cells. Subsequently, PDCD4 proteins were precipitated using streptavidin beads, and the ubiquitination level of PDCD4 was detected by western blotting with an anti-HA antibody. The result demonstrated that the overexpression of NPMc+ significantly increased the polyubiquitination of PDCD4 (Figure 4G). Collectively, these results imply that NPMc+ regulating the ubiquitination degradation of PDCD4 could be one of the mechanisms contributing to the progression of AML.

### Blockage of the interaction between NPMc+ and PDCD4 inhibits NPM1-mutated AML cell proliferation

**Figure 5:**
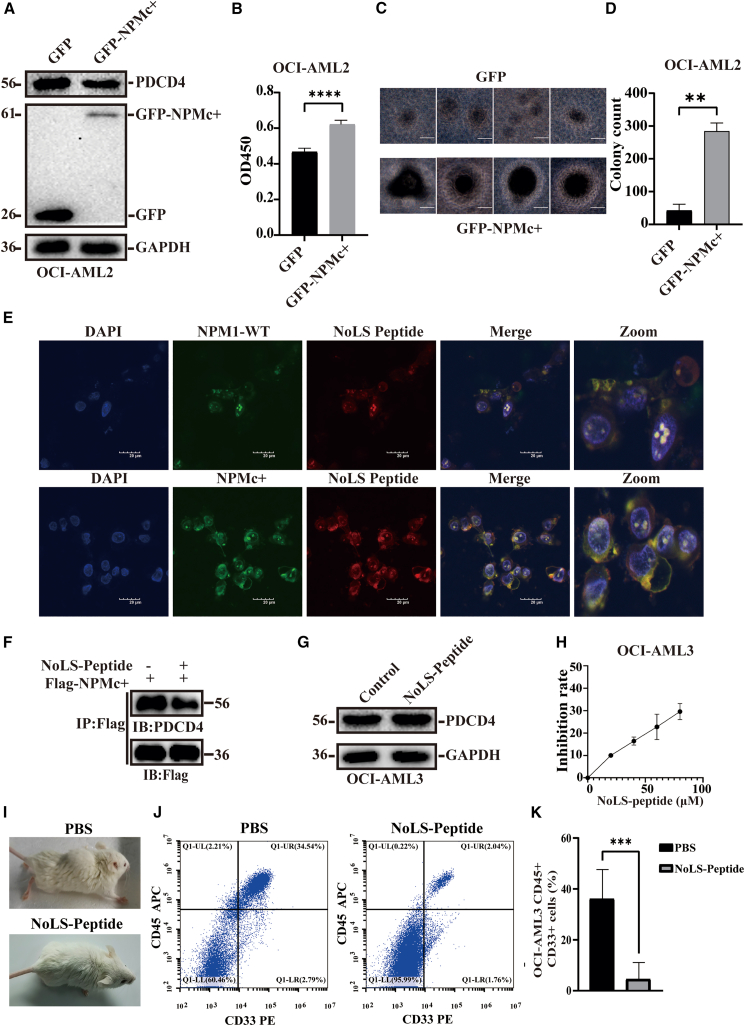
Blockage of the interaction between NPMc+ and PDCD4 inhibits NPM1-mutated AML cell proliferation (A) Western blotting detected the expression level of PDCD4 and GFP-NPMc+ in OCI-AML2 cells after electrotransfection. (B) CCK-8 assay showed that NPMc+ promoted the proliferation of AML cells. (C) Colony formation assay of the AML cells overexpression with GFP-NPMc+. Scale bar: 50 μm. (D) Histogram of colony inhibition rate. (E) Confocal microscopy to analyze the co-localization of GFP-NPMc+ with rhodamine-labeled NoLS peptides. Scale bar: 20 μm. (F) Co-immunoprecipitation of NPMc+ and PDCD4 with or without NoLS peptide treatment. (G) Western blotting analysis of the expression level of PDCD4 in AML cells treated with NoLS peptide. (H) CCK-8 assay showing the proliferation inhibition effect of NoLS peptide on OCI-AML3 cells. (I) A comparison of the hindlimb paralysis and hunched appearance in the control group and the NoLS peptide-treated group AML mouse group. (*n* = 5). (J) Flow cytometric to determine the percentage of leukemic cell engraftment in AML mouse models treated with NoLS peptides. (K) Quantitative analysis of OCI-AML3 cells in AML mouse. All data are presented as mean ± SD of three independent experiments. *p* value is calculated using the unpaired two-tailed *t* test. ∗∗*p* < 0.01; ∗∗∗*p* < 0.001 and ∗∗∗∗*p* < 0.0001.

Next, we aimed to determine whether NPMc+ regulated the expression of PDCD4 in AML cells and facilitated the proliferation of these cells. GFP and GFP-NPMc+ plasmids were electrotransfected into NPM1-wildtype AML cells, OCI-AML2. Western blotting analysis revealed that overexpression of NPMc+ led to a reduction in PDCD4 levels (Figure 5A). Moreover, the overexpression of NPMc+ promoted cell proliferation (Figure 5B) and enhanced colony formation in AML cells (Figures 5C and 5D). These findings indicated that the interaction between NPMc+ and PDCD4, which mediated the degradation of PDCD4, played a crucial role in the progression of AML. Thus, blocking the interaction between NPMc+ and PDCD4 could potentially be a viable therapeutic strategy for AML. To test this hypothesis, a NoLS peptide derived from the PDCD4-NoLS region (aa 53–73) was synthesized and introduced into cells. Rhodamine labeled NoLS peptide co-localized with GFP-NPM1 and GFP-NPMc+, suggesting that the NoLS peptide could competitively bind to NPM1 proteins (Figure 5E). Subsequently, we investigated whether the NoLS peptide could block the interaction between NPMc+ and PDCD4. As expected, the co-IP assay showed that Flag-NPMc+ interacted with PDCD4, but this binding was diminished when the cells were treated with the NoLS peptide (Figure 5F). Additionally, the expression level of PDCD4 increased in OCI-AML3 cells following treatment with the NoLS peptide, indicating that blockage of the interaction between NPMc+ and PDCD4 by the NoLS peptide inhibited the degradation of PDCD4 (Figure 5G). Next, we examined the proliferation inhibitory effect of the NoLS peptide on AML cells. The CCK-8 assay showed that the NoLS peptide inhibited the proliferation of OCI-AML3 cells in a dose-dependent manner (Figure 5H). Moreover, a mouse model of NPM1-mutated AML was established by injecting OCI-AML3 cells into the tail vein of NOG mice. After three weeks, the mice displayed hindlimb paralysis and a hunched appearance. These mice were then treated with the NoLS peptide for two weeks. Compared to the control group treated with PBS, the group treated with the NoLS peptide showed a significantly reduction in hindlimb paralysis and hunched appearance (Figure 5I). Subsequently, the mice were sacrificed, and bone marrow cells were isolated, stained with anti-human APC-CD45 and PE-CD33 antibodies, and analyzed by flow cytometry to determine leukemic cell engraftment. The NoLS peptide significantly inhibited the growth of OCI-AML3 cells in the mouse model (Figures 5J and 5K). Collectively, these results indicate that NPMc+ promotes the ubiquitination degradation of PDCD4, which is crucial for the progression of AML. Blocking the interaction between NPMc+ and PDCD4 using the PDCD4-NoLS peptide can effectively inhibit the proliferation of AML cells.

### PDCD4 interacts with RBBP4 in the nucleus

**Figure 6:**
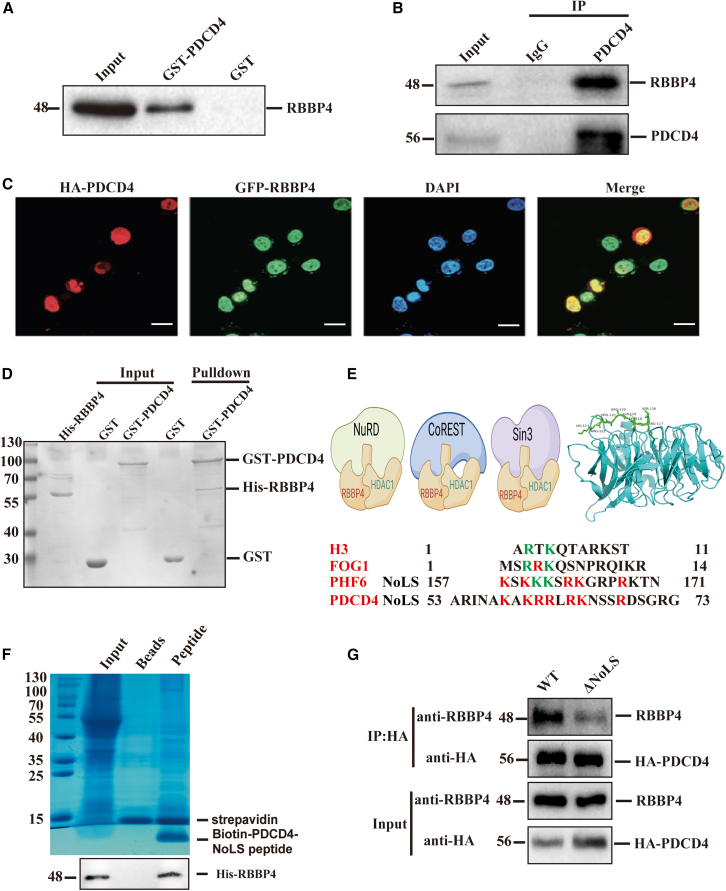
PDCD4 interacts with RBBP4 in the nucleus (A) Western blotting analysis using anti-RBBP4 antibody to detect the binding with PDCD4 in GST pull-down sample. (B) Co-immunoprecipitation of PDCD4 and RBBP4. IgG was used as negative control. (C) Co-localization of RBBP4 (green) and PDCD4 (red). Cell nuclei were counterstained with DAPI. Scale bar: 10 μm. (D) GST pull-down assay to detect the direct interaction between PDCD4 and RBBP4 using the purified proteins. (E) RBBP4 is a core component of HDAC complexes, including NuRD, CoREST, and Sin3. The H3, FOG1, or PHF6 NoLS peptide binds to the top surface of RBBP4 in a similar manner. Sequence alignment between PDCD4 NoLS and H3, FOG1, or PHF6 peptide reveals that the RBBP4-binding motif is highly conserved. (F) Biotin-pull down assay to detect the binding of PDCD4-NoLS peptide and RBBP4 protein. The bound proteins were subjected to SDS-PAGE and analyzed by western blotting. (G) Co-IP assay to detect the interaction between RBBP4 and NoLS-deletion mutant of PDCD4. HA-tagged PDCD4 or PDCD4-ΔNoLS proteins was overexpressed in HEK-293T cells, then cell lysate was immunoprecipitated with anti-HA beads, followed by western blot using anti-HA and anti-RBBP4 antibody.

PDCD4 is typically localized in the nucleus. To explore the significance of NPMc+-induced mislocation of PDCD4 in NPM1-mutated AML, we analyzed the interaction proteins of PDCD4 from mass spectrometry data. We identified RBBP4 as a crucial binding protein of PDCD4 in the nucleus. This was verified through western blotting analysis of the GST-PDCD4 pull-down sample, using an anti-RBBP4 antibody (Figure 6A). The Co-IP assay further detected the interaction between PDCD4 and RBBP4 (Figure 6B). Confocal microscopy revealed the co-localization of PDCD4 and RBBP4 within the nucleus (Figure 6C). Additionally, the GST pull-down assay demonstrated that the purified his-tagged RBBP4 protein directly interacted with GST-PDCD4 (Figure 6D, lane 5). These findings suggest that PDCD4 binds directly to RBBP4 and may be implicated in the function of RBBP4. Previous research has reported that RBBP4 is a component of multiple histone deacetylase (HDAC) complexes. These complexes are involved in chromatin remodeling, histone post-translational modifications and gene expression regulation. Such as the nucleosome remodeling and deacetylase (NuRD) complex, the HDAC-co-repressor of repressor element-1 silencing transcription factor (CoREST) complex and the switch independent 3 (Sin3) complex.^35^^,^^36^^,^^37^ Structural analysis of RBBP4 has shown that the protein forms a seven-bladed β-propeller. It contains two binding sites, a c-site on the top of the WD40 repeats and another on the side of the WD40 domain, between the N-terminal α-helix and PP-loop. Recently, the structures of several RBBP4 complex, such as RBBP4-PHF6, RBBP4-H3, and RBBP4-FOG1, have been determined. The interaction sites of these proteins share the same “RKK” motif.^38^^,^^39^^,^^40^ Sequence alignment indicated a high degree of sequence similarity between the NoLS of PDCD4 and those of other RBBP4-binding partners (Figure 6E). This suggests that the NoLS of PDCD4 may be directly involved in its interaction with RBBP4. To test this hypothesis, we performed a peptide pull-down assay. In this assay, a biotin-tagged PDCD4-NoLS peptide was immobilized on streptavidin-conjugated beads and incubated with AML cell lysate. As expected, RBBP4 was efficiently pulled down by the NoLS peptide, while no binding of RBBP4 was observed when no peptide was added (Figure 6F). To further confirm this interaction, we performed a Co-IP assay to detect the interaction between RBBP4 and a NoLS-deletion mutant of PDCD4. The result showed that the absence of NoLS affected the binding of PDCD4 to RBBP4 (Figure 6G).

### PDCD4 is involved in the regulation of NPMc+ mediated Hox gene expression

**Figure 7:**
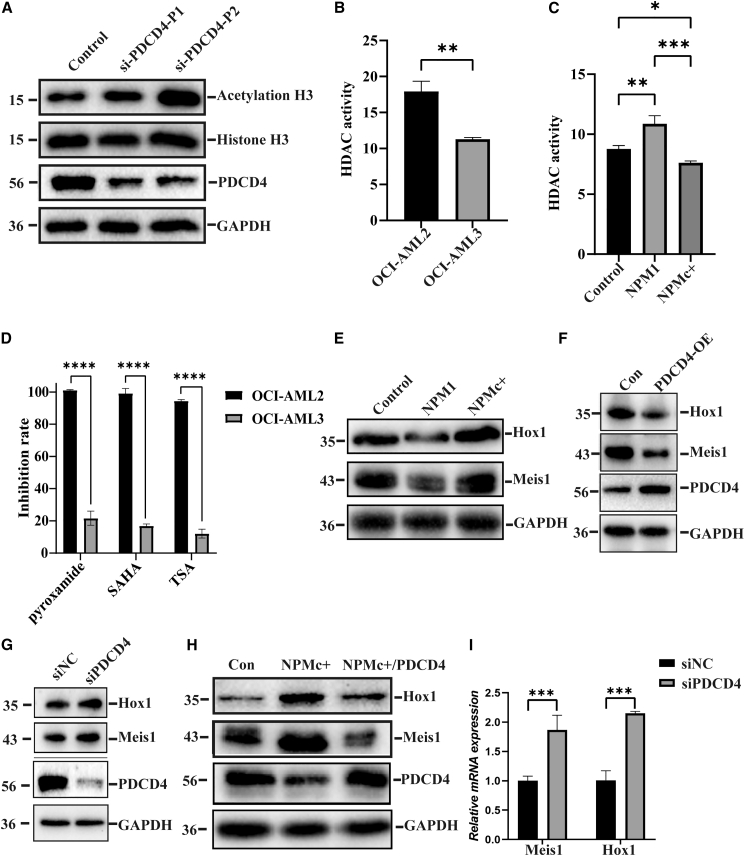
PDCD4 is involved in the regulation of NPMc+ mediated Hox gene expression (A) Western blotting detected the acetylation level of histones in the PDCD4-knockdown HEK293T cells. (B) HDAC activity in the OCI-AML2 and OCI-AML3 cells was determined using HDAC fluorometric activity assay kit (AAT Bioquest). (C) As in B, but HDAC activity was detected in the HEK293T cells overexpressing GFP, GFP-NPM1 or GFP-NPMc+. (D) CCK-8 assay showing the proliferation inhibition effect of HDAC inhibitors on AML cells. (E) Western blot analysis of the levels of Hox1 and Meis1 in HEK293T cells overexpressing GFP-NPM1 or GFP-NPMc+. (F) As in E, but HEK293T cells overexpressing PDCD4. (G) As in F, but in PDCD4-knockdown cells. (H) Western blotting detected the expression level of Hox1 and Meis in HEK293T cells overexpressing GFP-NPMc+ alone or co-expressing NPMc+ and PDCD4. (I) RT-qPCR analysis of mRNA levels of Hox1 and Meis1 gene in PDCD4-knockdown cells. All data are presented as mean ± SD of three independent experiments. *p* value is calculated using the unpaired two-tailed *t* test (two groups) and one-way ANOVA (more than two groups). ∗*p* < 0.05; ∗∗*p* < 0.01; ∗∗∗*p* < 0.001 and ∗∗∗∗*p* < 0.0001.

To determine whether PDCD4 regulated the histone deacetylation through interaction with RBBP4, a western blotting assay was carried out to detect the acetylation level of histones in PDCD4-knockdown HEK293T cells. The result showed that knocking down PDCD4 expression significantly increased histone acetylation levels. This finding suggested a synergistic effect between PDCD4 and RBBP4 on histone deacetylase (HDAC) activity (Figure 7A). Since NPMc+ induced the degradation of PDCD4, this could potentially lead to a decrease in HDAC activity in NPM1-mutated AML cells. Subsequently, we measured the HDAC activity in OCI-AML2 and OCI-AML3 cells. As expected, lower HDAC activity was detected in the NPM1-mutated AML cell line, OCI-AML3 cells (Figure 7B). We further examined the HDAC activity in HEK293T cells overexpressing NPM1 and NPMc+. Similarly, overexpression of NPMc+ inhibited HDAC activity (Figure 7C). Additionally, we treated OCI-AML2 and OCI-AML3 cells with three HDAC inhibitors: trichostatin A (TSA), suberoylanilide hydroxamic acid (SAHA), and pyroxamide. The CCK-8 assay showed that OCI-AML2 cells were highly sensitive to these HDAC inhibitors, while no obvious inhibitory effect was observed in OCI-AML3 cells (Figure 7D). These results imply that NPMc+ inhibits the function of HDAC complexes by reducing PDCD4 expression, which may be involved in the regulation of gene transcription. Recent studies have shown that NPMc+ directly modulates the expression of HOX gene and its cofactor MEIS1 by regulating transcriptional complexes. We also observed an increase in the expression levels of Hox1 and Meis1 in in cells overexpressing NPMc+ (Figure 7E). Next, we further investigated whether PDCD4 was involved in the regulation of Hox gene expression. Western blotting analysis revealed that overexpression of PDCD4 inhibited the expression level of Hox1 and Meis1 (Figure 7F). Conversely, knocking down of PDCD4 promoted the expression level of Hox1 and Meis1 (Figure 7G). Additionally, the increased expression of Hox1 and Meis1 induced by NPMc+ could be partially reversed by overexpressing PDCD4 (Figure 7H). Finally, we examined whether PDCD4 regulated the transcription of Hox1 and Meis1. As respected, knocking down PDCD4 significantly enhanced the transcription of Hox1 and Meis1 (Figure 7I). These results suggest that NPMc+ may regulate gene transcription by modulating the mislocalization and degradation of PDCD4.

## Discussion

In this study, we observed a marked reduction in PDCD4 protein expression in AML cell. To clarify the mechanism underlying the aberrant expression of PDCD4 in AML and its functional role in leukemogenesis, we conducted the GST pull-down assay and identified the interaction between NPM1 and PDCD4. NPM1 is a multifunctional protein with essential cellular functions. One of its key functions lies in ribosome biogenesis, where it facilitates interactions between ribosomal proteins and rRNA. A recent report indicated that PDCD4 directly interacted with ribosomes, suggesting that NPM1 could act as a chaperone for PDCD4 to bind to ribosomes.^41^ Multiple studies have determined that the N-terminal domain of NPM1 interacts with proteins by recognizing the putative NoLS sequences in its interactors. This recognition assists in their transport to, and localization within, the nucleus and nucleoli.^42^ Indeed, we identified a NoLS sequence at the N-terminus of PDCD4, which directly binds to the N-terminal domain of NPM1.

In numerous tumor types, alterations in NPM1 have been observed. One of the most well-known is the mutation of NPM1 in AML. The mutated NPM1, often denoted as NPMc+, is abnormally located in the cytoplasm, potentially playing a role in leukemogenesis. Since NPMc+ retains an intact N-terminal domain, it can still interact with PDCD4, causing PDCD4 to mislocalize to the cytosol. NPMc+ and PDCD4 exhibit strong co-localization in the cytoplasm, and the expression level of PDCD4 is significantly reduced in the NPM1-mutated cell line, OCI-AML3. Additionally, we discovered that NPMc+ directly regulates the activation of the AKT-mTOR signaling pathway mediated by PDCD4 (Figure S2). Further study demonstrated that NPMc+ induced the ubiquitination degradation of PDCD4, which is crucial for the progression of AML. Consequently, the NPM1 surface involved in the interaction with PDCD4 could potentially serve as a target for small molecules interference in the treatment of NPM1-mutated AML. In recent years, various therapeutic strategies targeting NPMc+ have been explored. NSC348884 disrupts NPM1 oligomerization and induces apoptosis in NPM1-mutated OCI-AML3 cells by targeting the N-terminal of NPM1.^43^ KPT-330 (selinexor) and KPT-8602 (eltanexor), inhibitors of the nuclear export protein XPO1, exhibit anti-leukemic activity by suppressing NPMc+ translocation.^44^ The combination of arsenic trioxide (ATO) and all-trans retinoic acid (ATRA) triggers proteasome-dependent degradation of NPMc+ and induces death in AML cells.^45^ BCL-2 inhibitor venetoclax and Menin inhibitors demonstrate strong anti-leukemic activity against NPM1-mutated AML cells. Additionally, targeting multiple signaling pathways and diverse immunotherapeutic approaches, including immune checkpoint inhibitors, as well as CAR and TCR T cell-based therapies have shown potent anti-leukemic effects.^46^^,^^47^ Furthermore, NPM1 interacts with numerous protein partners, primarily via its N-terminal domain. A large surface within NPM1’s N-terminal domain acts as a docking site for proteins bearing a NoLS motif.^48^ Consequently, disruption of these interactions has emerged as a viable strategy to inhibit the specific functions of NPM1 in cancer cells. The pseudopeptide N6L has been synthesized and has already completed Phase 1/2a clinical trials for various solid tumors. Composed of a multimeric pseudopeptide rich in positively charged residues, N6L shares properties similar to NoLS and has a high affinity for the N-terminal domain of NPM1. However, its inhibitory effect was relatively limited in OCI-AML3 cells. N6L significantly reduces OCI-AML2 cell growth by activating a p53-dependent apoptotic pathway, yet its effect is less pronounced in OCI-AML3 cells due to delayed p53 activation.^49^ In our study, we found that the synthesized PDCD4-NoLS peptide effectively blocked the interaction between NPMc+ and PDCD4. Moreover, it was able to exert an antiproliferative effect on NPMc+ positive AML cells both *in vitro* and *in vivo*. These findings suggest that PDCD4-NoLS peptide may hold promise as a potential therapeutic agent.

PDCD4 has predominantly nuclear localization, while numerous studies focus on the role of PDCD4-mediated translation inhibition. Although several translation targets of PDCD4 such as Sin1, p53, c-Myb, Bcl-XL, and XIAP have been identified, contributing to our understanding of its tumor-suppressing role, the function of nuclear PDCD4 remains elusive.^50^^,^^51^ NPMc+ causes the cytosolic delocalization and degradation of PDCD4, thereby disrupting its function in both the cytoplasm and nucleus. In this study, we have identified RBBP4 as the interaction partner of PDCD4. RBBP4 is a component of multiple histone deacetylase (HDAC) complexes and is involved in transcriptional regulation. Intriguingly, the NoLS sequence of PDCD4 is also involved in the interaction with RBBP4. Moreover, deletion of the NoLS domain in the PDCD4 protein leads to the loss of its tumor-suppressive function (Figure S3). In addition, knocking down PDCD4 expression significantly increases histone acetylation levels. This indicates that PDCD4 may be directly involved in the assembly of HDAC complexes and transcriptional regulation. Since NPMc+ induces the degradation of PDCD4, this could potentially result in a decrease in HDAC activity in NPM1-mutated AML cells. In fact, lower HDAC activity was detected in the NPM1-mutated AML cell line, OCI-AML3 compared to the NPM1-wildtype OCI-AML2 cells. Additionally, no obvious inhibitory effect of HDAC inhibitors was observed in OCI-AML3 cells. This may account for the ineffectiveness of HDAC inhibitors in AML clinical trials. Recent studies have shown that NPMc+ directly modulates the transcription of HOX1 gene to maintain the leukemic state.^52^ We discovered that PDCD4 is associated with the transcriptional regulation of the HOX1 gene and its cofactor MEIS1. Interestingly, we also found that reducing PDCD4 promotes NPM1 expression, suggesting a positive regulatory feedback loop between NPMc+ and PDCD4 (Figure S4). Collectively, NPMc+ may induce leukemia at both the transcription and translation level by regulating mislocalization and degradation of PDCD4, and disrupting its function in the cytoplasm and nucleus. Blocking the interaction between NPMc+ and PDCD4 using a PDCD4-driven NoLS peptide could be a promising therapeutic approach for treating NPM1-mutated AML.

### Limitations of the study

Although this study provides key insights into the pathogenic mechanism of NPM1-mutated AML and suggests the NPMc+/PDCD4 complex could be a potential therapeutic target for this subtype of AML, several limitations should be acknowledged. In this study, although we found that NPMc+ interacts with PDCD4 regulating the HDAC activity and histone acetylation at the cellular level, it has not been validated in animal models and clinical samples. Additionally, we did not investigate the relationship between PDCD4 and NPM1 wild-type AML. The detailed mechanism of PDCD4 in NPM1 wild-type AML needs to be further investigated in future study.

## Resource availability

### Lead contact

Further information and requests for resources and reagents should be directed to and will be fulfilled by the lead contact, Fuyun Wu (wufuyun100@126.com).

### Materials availability

This study did not generate new unique reagents.

### Data and code availability

All data reported in this paper will be shared by the lead contact upon request. This paper does not report original code. Any additional information required to reanalyze the data reported in this paper is available from the lead contact upon request.

## Acknowledgments

We thank Biomedical Research Institute of Hubei University of Medicine for providing technical support. This work was supported by the 10.13039/501100003819Hubei Provincial Natural Science Foundation (grant no. 2023AFB882). The Joint Funds of the 10.13039/501100003819Hubei Provincial Natural Science Foundation (JCZRLH202500273). Innovative Research Program for Graduates of 10.13039/501100014361Hubei University of Medicine (grant no. JC2025005). The Training Program of Innovation and Entrepreneurship for Undergraduates (grant no. X202410929074).

## Author contributions

Conceptualization, F.W., S.L., and D.L.; methodology, C.L. and J.K.; investigation, C.L., J.K., Z.Z., H.G., and H.S.; writing—original draft, S.L. and D.L.; writing—review & editing, F.W.; funding acquisition, Z.Z., H.S., F.W., and S.L.; resources, D.L. and H.G.; supervision, F.W. and S.L.

## Declaration of interests

The authors declare no competing interests.

## STAR★Methods

### Key resources table

**Table:** 

REAGENT or RESOURCE	SOURCE	IDENTIFIER
**Antibodies**		

anti-NPM1	Proteintech	Cat#: 60096-1-Ig; RRID: AB_2155162
anti-PDCD4	Proteintech	Cat#: 66100-1-Ig; RRID: AB_2881499
anti-GFP	Proteintech	Cat#: 66002-1-Ig; RRID: AB_11182611
anti-GAPDH	Proteintech	Cat#: 10494-1-AP; RRID: AB_2263076
anti-Flag	Proteintech	Cat#: 20543-1-AP; RRID: AB_11232216
anti-HA	MBL	Cat#: M180-3; RRID: AB_10951811
PE anti-human CD33	BioLegend	Cat#: 366607; RRID: AB_2566106
APC anti-human CD45	BioLegend	Cat#: 304011; RRID: AB_314399

**Bacterial and virus strains**		

DH5α Chemically Competent Cell	Beyotime	Cat#: D1031S
BL21 Chemically Competent Cell	Beyotime	Cat#: D1009S
pcSLenti-EF1-EGFP-F2A-Puro-CMV-NPM1-Flag	This paper	N/A
pcSLenti-EF1-EGFP-F2A-Puro-CMV-NPMc+-Flag	This paper	N/A

**Biological samples**		

Human PBMCs	This paper	N/A

**Chemicals, peptides, and recombinant proteins**		

RPMI 1640	Gibco	Cat#: 11875093
Lipo8000	Beyotime	Cat#: C0533
CHX	MCE	Cat#: HY-12320
MG132	MCE	Cat#: HY-13259
Streptactin Beads 4FF	Biodragon	Cat#: BDTL0026
Glutathione Sepharoe 4FF	GE healthcare	Cat#:17513201
Ni Sepharose	GE healthcare	Cat#:17526801
Biotin-PDCD4-NoLS peptide: Biotin- EARINAKAKRRLRKNSSRDSGRG	GL Biochem	N/A
TAT-PDCD4-NoLS peptide: YGRKKRRQRRRGGEARINAKAKRRLRKNSSRDSGRG	GL Biochem	N/A
Rhodamine-TAT-PDCD4-NoLS peptide	GL Biochem	N/A
GST-PDCD4 recombinant proteins	This paper	N/A
His-NPM1 recombinant proteins	This paper	N/A

**Critical commercial assays**		

BCA Protein Assay Kit	Thermo Fisher	Cat#: 23225
ChamQ Universal SYBR qPCR Master Mix	Vazyme	Cat#: Q711-02
Human Neutrophil Isolation Kit	Solarbio	Cat#: P9040
FastPure EndoFree Plasmid Maxi Kit	Vazyme	Cat#: DC202
Dynabeads™ Co-Immunoprecipitation Kit	Invitrogen	Cat#: 14321D

**Experimental models: Cell lines**		

OCI-AML2	zqxzbio	Cat#: ZQ0919
OCI-AML3	zqxzbio	Cat#: ZQ0580
THP-1	zqxzbio	Cat#: ZQ0086

**Experimental models: Organisms/strains**		

NOG/SCID mice	Wei-tong Lihua Experimental Animal Technical Company	N/A

**Oligonucleotides**		

siRNA targeting sequence PDCD4-P1: 5′-GCGGAAAUGUUAAGAGAUU-3′ PDCD4-P2: 5′-GCACAACUGAUGUGGAAAA-3′	Tsingke	N/A
PDCD4 primer (forward) 5′- ACTGTGCCAACCAGTCCAAAGG -3′	Tsingke	N/A
PDCD4 primer (reverse) 5′- CCTCCACATCATACACCTGTCC-3′	Tsingke	N/A
β-actin primer (forward) 5′- GTACCACTGGCATCGTGATG -3′	Tsingke	N/A
β-actin primer (reverse) 5′- CCGCTCATTGCCAATGGTGAT -3′	Tsingke	N/A
HOX1 primer (forward) 5′- GCTTGTGGTTCTCCTCCAG -3′	Tsingke	N/A
HOX1 primer (reverse) 5′- TCCCTGGTGAGGTACATGT -3′	Tsingke	N/A
Meis1 primer (forward) 5′- CTGTTTGAAAGGGAAAAT -3′	Tsingke	N/A
Meis1 primer (reverse) 5′- TGGAAGGGCCTGGGGTT -3′	Tsingke	N/A

**Recombinant DNA**		

Plasmid: GFP-NPM1, GFP-NPMc+	This paper	N/A
Plasmid: GFP-PDCD4, GST-PDCD4	This paper	N/A
Plasmid: His-NPM1, His-NPM (1-117,118-188,189-294)	This paper	N/A

**Software and algorithms**		

GraphPad Prism v9.3	GraphPad	N/A
ImageJ	ImageJ	N/A
FlowJo	BD Bioscience	N/A

**Other**		

NanoTemper/Monolith NT.115	NanoTemper	N/A
LSM510 META Confocal Imaging System	Zeiss	N/A

### Experimental model and study participant details

#### Human PBMC

The peripheral blood samples from 4 newly diagnosed AML patients and 3 healthy donors were collected from the affiliated hospital of Hubei University of Medicine. The clinic pathological characteristics of AML patients were provided in Table S1. Prior to their participation in the study, informed consent was obtained from all patients. Mononuclear cells were isolated from the peripheral blood samples by Ficoll-Paque density gradient centrifugation, then the cell samples were subjected to SDS-PAGE. The study protocol was approved by the ethics committee of Hubei University of Medicine (No. 2023-EER-11).

#### Animal

NOG/SCID mice (female, 6 weeks old) were obtained from Wei-tong Lihua Experimental Animal Technical Company (Beijing, China). The mice were kept under specific pathogen-free conditions. They were exposed to a 12-hour light-dark cycle and had access to plentiful food and water. The animal study was approved by the Hubei University of Medicine Animal Ethics Committee (No. 2023104).

#### Cell lines

Human acute myeloid leukemia cell lines THP-1/OCI-AML2/OCI-AML3 were obtained from the Shanghai Zhong Qiao Xin Zhou Biotechnology Co., Ltd. (Shanghai, China). All the cell lines were authenticated by short tandem repeat (STR) profiling and tested for mycoplasma contamination.

### Method details

#### Plasmids and peptides

The human NPM1 and PDCD4 gene were amplified by PCR from the cDNA of HEK293T cells and cloned into the pEGFP-N1 vector. The NPM1 fragments were cloned into the pET-28a vector to generate His-NPM1 fragments. PDCD4 was cloned into the pGEX-6P-1 vector to generate GST-PDCD4. Biotin-labeled PDCD4-NoLS peptide and TAT-PDCD4-NoLS peptide were purchased from GL Biochem (Shanghai, China).

#### Cell culture and transfection

THP-1/OCI-AML2/OCI-AML3 were cultured in RPMI 1640 medium supplemented 10% fetal bovine serum. To achieve stable expression of NPM1 or NPMc+, the NPM1 or NPMc+ gene was cloned into the lenti-CMV vector for lentivirus packaging. Cells were incubated with viruses for 48h and then selected with puromycin. The AML cells were electrotransfected with different plamids or siRNAs using Gene Pulser Xcell Total System (Bio-Rad). The sequences of siRNA for PDCD4 are as follows: si-PDCD4-P1 sence (5′-GCGGAAAUGUUAAGAGAUU -3′). si PDCD4-P2:(5′-GCACAACUGAUGUGGAAAA-3′).

#### Protein expression

The E. coli strain BL21 (DE3) cells harboring the expression plasmid pET-28a-NPM1 and GST-PDCD4 were cultured in LB medium at 37 °C until the optical density at 600 nm (OD600) reached 0.8, then induced with 0.5 mM IPTG at 24°C for 12h. The cells were harvested and lysed by sonication. The his-NPM1 proteins were purified using Ni–NTA chromatography and the GST-PDCD4 proteins were purified with Glutathione Sepharose 4FF (GE healthcare).

#### Pull down assay

GST-PDCD4 protein were immobilized on Glutathione Sepharose 4FF beads and then incubated with cell lysates or purified His-tagged NPM1 fragment proteins. After incubation, the beads were washed and resuspended in 2× SDS loading buffer. Following a 10-minute incubation at 95 °C, the samples were analyzed by SDS-PAGE gels and Coomassie blue staining.

#### Western blotting

The whole cell lysates were extracted using RIPA buffer, and the protein concentration was determined via BCA assay. Samples were resolved by SDS-PAGE and analyzed by standard Western blotting techniques. The following antibodies, all purchased from Proteintech, were utilized: rabbit polyclonal antibodies against GAPDH (10494-1-AP), Flag (20543-1-AP); Mouse monoclonal antibody against NPM1(60096-1-Ig), PDCD4 (66100-1-Ig), GFP (66002-1-Ig). The antibody against HA (M180-3) was purchased from MBL. The antibody against strep (bsm-33016M) was purchased from Bioss (China).

#### RT-qPCR

Total RNA was extracted from AML cells using TRIzol reagent (Vazyme, China), Subsequently, the RNA was reverse-transcribed to cDNA with the HiScript Reverse Transcriptase Kit (Vazyme, China) and qPCR was carried out using ChamQ Universal SYBR qPCR Master Mix (Vazyme China) on the CFX96 Touch Real-Time PCR Detection System. β-actin was used as an internal reference gene and the relative gene expression was determined using the 2−ΔΔCT method. The primer sequences are as follows: PDCD4-forward (5′- ACTGTGCCAACCAGTCCAAAGG -3′), PDCD4-reverse (5′- CCTCCACATCATACACCTGTCC-3′). β-actin forward (5′- GTACCACTGGCATCGTGATG -3′) and β-actin reverse (5′- CCGCTCATTGCCAATGGTGAT -3′). HOX1 forward (5′- GCTTGTGGTTCTCCTCCAG -3′) and HOX1 reverse (5′- TCCCTGGTGAGGTACATGT -3′). Meis1 forward (5′- CTGTTTGAAAGGGAAAAT -3′) and Meis1 reverse (5′- TGGAAGGGCCTGGGGTT -3′).

#### Immunofluorescence

OCI-AML2 and OCI-AML3 cell slides were prepared using the cytospin technique. HEK293T cells, after transfection, were seeded onto confocal glass-bottomed dishes. The cells were fixed with 4% paraformaldehyde for 30 minutes, permeabilized with 0.2% Triton X-100 for 10 minutes, and blocked with 3% BSA for 30 minutes. Subsequently, the cells were incubated with primary antibodies at 4°C overnight. The slides were then washed with PBS and incubated with fluorescent dye-conjugated secondary antibodies. The cell nuclei were counterstained with DAPI for 10 minutes. Immunofluorescence images were captured and analyzed using a Confocal laser scanning microscope (Zeiss, LSM510 META).

#### MicroScale thermophoresis (MST) assay

HEK293T cells transfected with GFP-PDCD4-FL, GFP-PDCD4-NT, or GFP-PDCD4-ΔNT were lysed using RIPA lysate without PMSF. 10 μL of the 50 μM purified NPM1-NT (aa 1-117) proteins were diluted 1:1 in 10 μL of buffer to make a 16-sample dilution series. The cell lysate was mixed with each ligand dilution and incubated for 10 minutes, then the mixtures were loaded into Standard Monolith Capillaries. Thermophoresis measurements were carried out using a Monolith NT.115 instrument (NanoTemper Technologies).

#### Ubiquitination assay

HEK293T cells stably expressing NPM1 or NPMc+ were co-transfected with HA-tagged ubiquitination (ub-HA) and strep-tagged PDCD4 (PDCD4-strep). After transfection, the cells were treated with 10 μM MG132 for 6 hours. Subsequently, the strep-tagged PDCD4 was pulled down using streptavidin beads. Ubiquitinated PDCD4 was detected and visualized by western blotting using both antibodies against HA and strep.

#### *In vivo* model of AML

NOG/SCID mice (female, 6 weeks old) were obtained from Wei-tong Lihua Experimental Animal Technical Company (Beijing, China). OCI-AML3 cells (3 × 10ˆ6) were injected into the tail vein of the NOG mice, which were then monitored daily for signs of leukemia. Ten of these mice were randomly divided into two groups and treated with PBS (control) or PDCD4-NoLS peptide (60 mg/kg, administered intraperitoneally once daily for 2 weeks). Mice were humanely sacrificed when they showed signs of distress, such as a hunched back or hind limb paralysis. Bone marrow cells were then isolated and stained with anti-human APC-CD45 and PE-CD33 antibodies. Flow cytometry was employed to determine the percentage of leukemic cell engraftment.

### Quantification and statistical analysis

Statistical significance was assessed using data derived from at least three independent experiments. Unpaired *t* test or one-way ANOVA were utilized for statistical analysis. The statistical method employed is specified in the corresponding figure legend. Data are presented as the mean ± SD. For all statistical tests, P < 0.05 was considered statistically significant. All the statistical plots were plotted using GraphPad Prism v.8.0 software.

### Additional resources

This study did not generate additional data.

## Supplemental information

Document S1. Figures S1–S4 and Data S1

Table S1. Clinical characteristics of newly diagnosed patients, related to Figure 1

## References

[1] Cui H., Wang Q., Lei Z., Feng M., Zhao Z., Wang Y., Wei G. (2019). DTL promotes cancer progression by PDCD4 ubiquitin-dependent degradation. J. Exp. Clin. Cancer Res..

[2] Wang Q., Yang H.S. (2018). The role of Pdcd4 in tumor suppression and protein translation. Biol. Cell.

[3] Li C., Du L., Ren Y., Liu X., Jiao Q., Cui D., Wen M., Wang C., Wei G., Wang Y. (2019). SKP2 promotes breast cancer tumorigenesis and radiation tolerance through PDCD4 ubiquitination. J. Exp. Clin. Cancer Res..

[4] Brito Querido J., Sokabe M., Díaz-López I., Gordiyenko Y., Zuber P., Du Y., Albacete-Albacete L., Ramakrishnan V., Fraser C.S. (2024). Human tumor suppressor protein Pdcd4 binds at the mRNA entry channel in the 40S small ribosomal subunit. Nat. Commun..

[5] Frankel L.B., Christoffersen N.R., Jacobsen A., Lindow M., Krogh A., Lund A.H. (2008). Programmed cell death 4 (PDCD4) is an important functional target of the microRNA miR-21 in breast cancer cells. J. Biol. Chem..

[6] Hu M., Lu Y., Zeng H., Zhang Z., Chen S., Qi Y., Xu Y., Chen F., Tang Y., Chen M. (2021). MicroRNA-21 maintains hematopoietic stem cell homeostasis through sustaining the nuclear factor-B signaling pathway in mice. Haematologica.

[7] Riccioni R., Lulli V., Castelli G., Biffoni M., Tiberio R., Pelosi E., Lo-Coco F., Testa U. (2015). miR-21 is overexpressed in NPM1-mutant acute myeloid leukemias. Leuk. Res..

[8] Li C., Yan H., Yin J., Ma J., Liao A., Yang S., Wang L., Huang Y., Lin C., Dong Z. (2019). MicroRNA-21 promotes proliferation in acute myeloid leukemia by targeting Krüppel-like factor 5. Oncol. Lett..

[9] Tang H., Chen Y., Zhang N., Deng J., Zhou K. (2023). Higher expression of programmed cell death 4 (PDCD4) in acute myeloid leukemia is associated with better prognosis after chemotherapy. Ann. Hematol..

[10] Matsuhashi S., Manirujjaman M., Hamajima H., Ozaki I. (2019). Control Mechanisms of the Tumor Suppressor PDCD4: Expression and Functions. Int. J. Mol. Sci..

[11] Wu P., Wang J., Mao X., Xu H., Zhu Z. (2021). PDCD4 regulates apoptosis in human peritoneal mesothelial cells and promotes gastric cancer peritoneal metastasis. Histol. Histopathol..

[12] Lu K., Chen Q., Li M., He L., Riaz F., Zhang T., Li D. (2020). Programmed cell death factor 4 (PDCD4), a novel therapy target for metabolic diseases besides cancer. Free Radic. Biol. Med..

[13] Suzuki C., Garces R.G., Edmonds K.A., Hiller S., Hyberts S.G., Marintchev A., Wagner G. (2008). PDCD4 inhibits translation initiation by binding to eIF4A using both its MA3 domains. Proc. Natl. Acad. Sci. USA.

[14] Chang J.H., Cho Y.H., Sohn S.Y., Choi J.M., Kim A., Kim Y.C., Jang S.K., Cho Y. (2009). Crystal structure of the eIF4A–PDCD4 complex. Proc. Natl. Acad. Sci. USA.

[15] Zhang Q., Peng J., Zhang Y., Liu J., He D., Zhao Y., Wang X., Li C., Kong Y., Wang R. (2025). The kinase PLK1 promotes Hedgehog signaling–dependent resistance to the antiandrogen enzalutamide in metastatic prostate cancer. Sci. Signal..

[16] Scholl C., Gilliland D.G., Fröhling S. (2008). Deregulation of Signaling Pathways in Acute Myeloid Leukemia. Semin. Oncol..

[17] Carter J.L., Hege K., Yang J., Kalpage H.A., Su Y., Edwards H., Hüttemann M., Taub J.W., Ge Y. (2020). Targeting multiple signaling pathways: the new approach to acute myeloid leukemia therapy. Signal Transduct. Target. Ther..

[18] Naji N.S., Sathish M., Karantanos T. (2024). Inflammation and Related Signaling Pathways in Acute Myeloid Leukemia. Cancers (Basel).

[19] Shiota M., Izumi H., Tanimoto A., Takahashi M., Miyamoto N., Kashiwagi E., Kidani A., Hirano G., Masubuchi D., Fukunaka Y. (2009). Programmed Cell Death Protein 4 Down-regulates Y-Box Binding Protein-1 Expression via a Direct Interaction with Twist1 to Suppress Cancer Cell Growth. Cancer Res..

[20] Hwang S.-K., Baker A.R., Young M.R., Colburn N.H. (2014). Tumor suppressor PDCD4 inhibits NF-κB-dependent transcription in human glioblastoma cells by direct interaction with p65. Carcinogenesis.

[21] Wang Q., Sun Z., Yang H.S. (2008). Downregulation of tumor suppressor Pdcd4 promotes invasion and activates both β-catenin/Tcf and AP-1-dependent transcription in colon carcinoma cells. Oncogene.

[22] Box J.K., Paquet N., Adams M.N., Boucher D., Bolderson E., O’Byrne K.J., Richard D.J. (2016). Nucleophosmin: from structure and function to disease development. BMC Mol. Biol..

[23] Okuwaki M. (2008). The Structure and Functions of NPM1/Nucleophsmin/B23, a Multifunctional Nucleolar Acidic Protein. J. Biochem..

[24] Gallo A., Lo Sterzo C., Mori M., Di Matteo A., Bertini I., Banci L., Brunori M., Federici L. (2012). Structure of Nucleophosmin DNA-binding Domain and Analysis of Its Complex with a G-quadruplex Sequence from the c-MYC Promoter. J. Biol. Chem..

[25] Grisendi S., Mecucci C., Falini B., Pandolfi P.P. (2006). Nucleophosmin and cancer. Nat. Rev. Cancer.

[26] Sekhar K.R., Freeman M.L. (2023). Nucleophosmin Plays a Role in Repairing DNA Damage and Is a Target for Cancer Treatment. Cancer Res..

[27] Grisendi S., Bernardi R., Rossi M., Cheng K., Khandker L., Manova K., Pandolfi P.P. (2005). Role of nucleophosmin in embryonic development and tumorigenesis. Nature.

[28] Shi Y., Xue Y., Wang C., Yu L. (2022). Nucleophosmin 1: from its pathogenic role to a tantalizing therapeutic target in acute myeloid leukemia. Hematology.

[29] Falini B., Brunetti L., Sportoletti P., Martelli M.P. (2020). *NPM1*-mutated acute myeloid leukemia: from bench to bedside. Blood.

[30] Wang A.J., Han Y., Jia N., Chen P., Minden M.D. (2020). NPM1c impedes CTCF functions through cytoplasmic mislocalization in acute myeloid leukemia. Leukemia.

[31] Falini B., Sorcini D., Perriello V.M., Sportoletti P. (2025). Functions of the native NPM1 protein and its leukemic mutant. Leukemia.

[32] Heath E.M., Chan S.M., Minden M.D., Murphy T., Shlush L.I., Schimmer A.D. (2017). Biological and clinical consequences of NPM1 mutations in AML. Leukemia.

[33] Brunetti L., Gundry M.C., Sorcini D., Guzman A.G., Huang Y.H., Ramabadran R., Gionfriddo I., Mezzasoma F., Milano F., Nabet B. (2018). Mutant NPM1 Maintains the Leukemic State through HOX Expression. Cancer Cell.

[34] Wang X.Q.D., Fan D., Han Q., Liu Y., Miao H., Wang X., Li Q., Chen D., Gore H., Himadewi P. (2023). Mutant NPM1 Hijacks Transcriptional Hubs to Maintain Pathogenic Gene Programs in Acute Myeloid Leukemia. Cancer Discov..

[35] Millard C.J., Varma N., Saleh A., Morris K., Watson P.J., Bottrill A.R., Fairall L., Smith C.J., Schwabe J.W.R. (2016). The structure of the core NuRD repression complex provides insights into its interaction with chromatin. eLife.

[36] Zhan Y., Yin A., Su X., Tang N., Zhang Z., Chen Y., Wang W., Wang J. (2024). Interpreting the molecular mechanisms of RBBP4/7 and their roles in human diseases (Review). Int. J. Mol. Med..

[37] Asmamaw M.D., He A., Zhang L.-R., Liu H.-M., Gao Y. (2024). Histone deacetylase complexes: Structure, regulation and function. Biochim. Biophys. Acta. Rev. Cancer.

[38] Liu Z., Li F., Zhang B., Li S., Wu J., Shi Y. (2015). Structural Basis of Plant Homeodomain Finger 6 (PHF6) Recognition by the Retinoblastoma Binding Protein 4 (RBBP4) Component of the Nucleosome Remodeling and Deacetylase (NuRD) Complex. J. Biol. Chem..

[39] Zhang W., Tyl M., Ward R., Sobott F., Maman J., Murthy A.S., Watson A.A., Fedorov O., Bowman A., Owen-Hughes T. (2013). Structural plasticity of histones H3-H4 facilitates their allosteric exchange between RbAp48 and ASF1. Nat. Struct. Mol. Biol..

[40] Cai L., Liu B., Cao Y., Sun T., Li Y. (2023). Unveiling the molecular structure and role of RBBP4/7: implications for epigenetic regulation and cancer research. Front. Mol. Biosci..

[41] Ye X., Huang Z., Li Y., Wang M., Meng W., Miao M., Cheng J. (2024). Human tumor suppressor PDCD4 directly interacts with ribosomes to repress translation. Cell Res..

[42] Di Matteo A., Franceschini M., Paiardini A., Grottesi A., Chiarella S., Rocchio S., Di Natale C., Marasco D., Vitagliano L., Travaglini-Allocatelli C., Federici L. (2017). Structural investigation of nucleophosmin interaction with the tumor suppressor Fbw7γ. Oncogenesis.

[43] Balusu R., Fiskus W., Rao R., Chong D.G., Nalluri S., Mudunuru U., Ma H., Chen L., Venkannagari S., Ha K. (2011). Targeting levels or oligomerization of nucleophosmin 1 induces differentiation and loss of survival of human AML cells with mutant NPM1. Blood.

[44] Bhatnagar B., Zhao Q., Mims A.S., Vasu S., Behbehani G.K., Larkin K., Blachly J.S., Blum W., Klisovic R.B., Ruppert A.S. (2020). Selinexor in Combination with Decitabine in Patients with Acute Myeloid Leukemia: Results from a Phase 1 study. Leuk. Lymphoma.

[45] El Hajj H., Dassouki Z., Berthier C., Raffoux E., Ades L., Legrand O., Hleihel R., Sahin U., Tawil N., Salameh A. (2015). Retinoic acid and arsenic trioxide trigger degradation of mutated NPM1, resulting in apoptosis of AML cells. Blood.

[46] Ranieri R., Pianigiani G., Sciabolacci S., Perriello V.M., Marra A., Cardinali V., Pierangeli S., Milano F., Gionfriddo I., Brunetti L. (2022). Current status and future perspectives in targeted therapy of NPM1-mutated AML. Leukemia.

[47] Florio D., Marasco D. (2024). Could Targeting NPM1c+ Misfolding Be a Promising Strategy for Combating Acute Myeloid Leukemia?. Int. J. Mol. Sci..

[48] Cela I., Di Matteo A., Federici L. (2020). Nucleophosmin in Its Interaction with Ligands. Int. J. Mol. Sci..

[49] De Cola A., Franceschini M., Di Matteo A., Colotti G., Celani R., Clemente E., Ippoliti R., Cimini A.M., Dhez A.C., Vallée B. (2018). N6L pseudopeptide interferes with nucleophosmin protein-protein interactions and sensitizes leukemic cells to chemotherapy. Cancer Lett..

[50] Wang Q., Zhu J., Wang Y.-W., Dai Y., Wang Y.L., Wang C., Liu J., Baker A., Colburn N.H., Yang H.-S. (2017). Tumor suppressor Pdcd4attenuates Sin1 translation to inhibit invasion in colon carcinoma. Oncogene.

[51] Liwak U., Thakor N., Jordan L.E., Roy R., Lewis S.M., Pardo O.E., Seckl M., Holcik M. (2012). Tumor Suppressor PDCD4 Represses Internal Ribosome Entry Site-Mediated Translation of Antiapoptotic Proteins and Is Regulated by S6 Kinase 2. Mol. Cell Biol..

[52] Khan I., Amin M.A., Eklund E.A., Gartel A.L. (2024). Regulation of HOX gene expression in AML. Blood Cancer J..

