# Supplementary material for: Mutant NPM1 modulates PDCD4 ubiquitination degradation and facilitates leukemogenesis
Source: iScience. 2025 Oct 15;28(11):113776. doi: 10.1016/j.isci.2025.113776 (PMC12604968; doi:10.1016/j.isci.2025.113776)
Supplement: Document S1. Figures S1–S4 and Data S1 [file mmc1.pdf]

iScience, Volume *28*

Supplemental information

Mutant NPM1 modulates PDCD4 ubiquitination degradation and facilitates leukemogenesis

Chuangxuan Liang, Jing Ke, Zhenyu Zhang, Huarong Guo, Hongxin Shang, Danwen Liu, Shan Li, and Fuyun Wu

Fig. S1

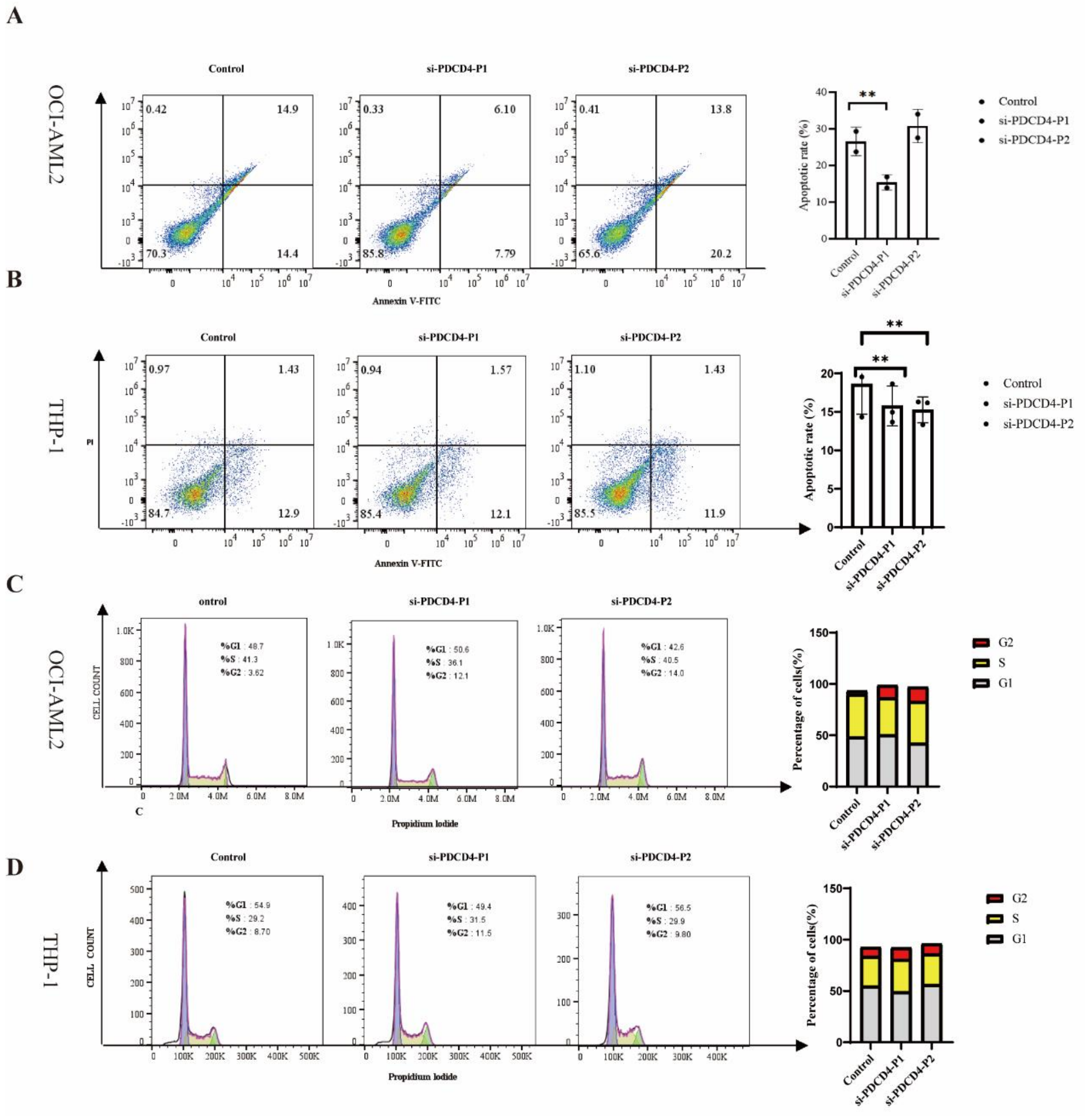

Fig. S1 Knockdown of PDCD4 reduced apoptosis and accelerated cell cycle progression in AML cells. (A) Flow cytometry analysis of apoptosis in OCI-AML2 cells with PDCD4 knockdown. (B) As in A, but in THP-1 cells. (C) Flow cytometry analysis of cell cycle in OCI-AML2 cells with PDCD4 knockdown. (D) As in C, but in THP-1 cells. All data are presented as mean ± SD of three independent experiments. p value is calculated using one-way ANOVA. $^{**}p < 0.01$.

Fig. S2

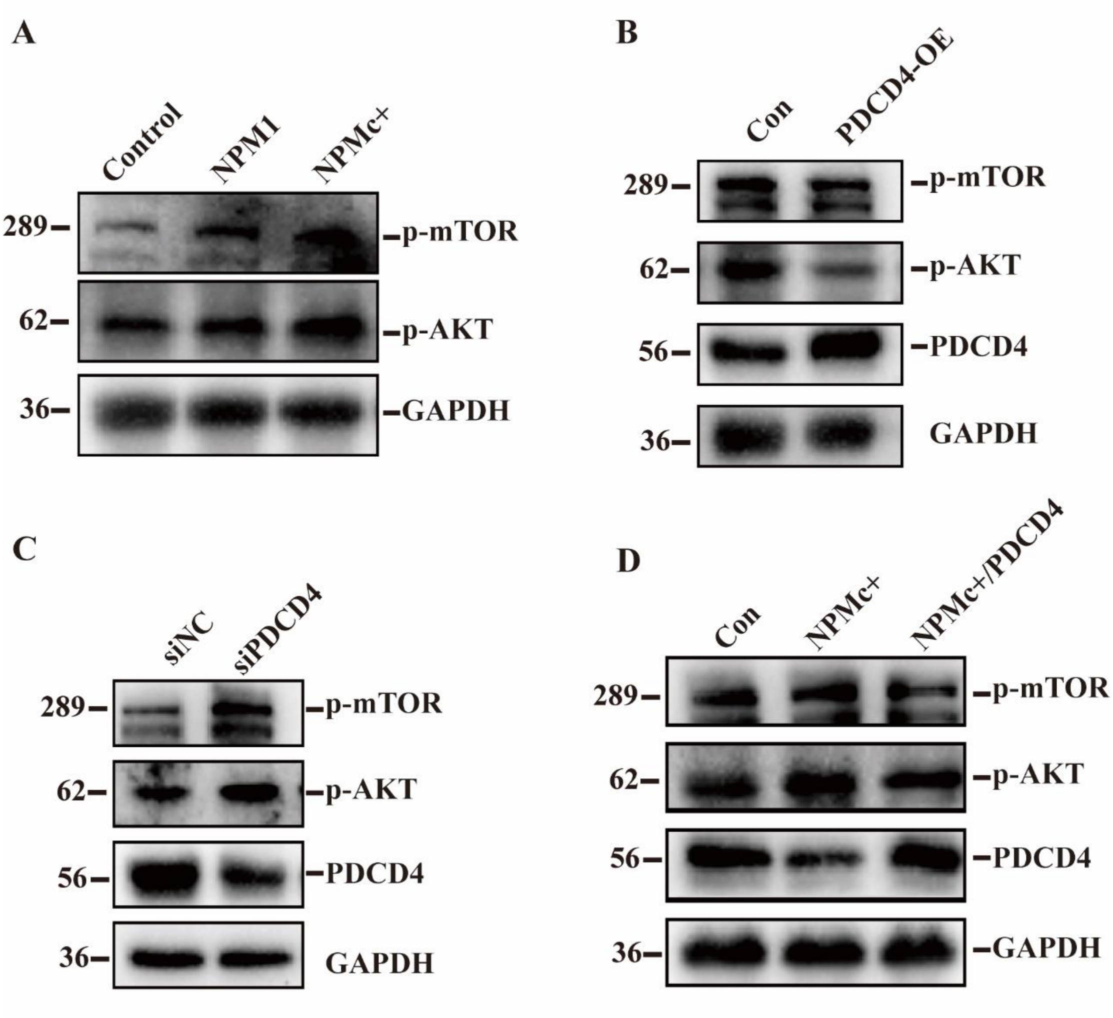

Fig. S2 NPMc+ regulates the activation of the AKT-mTOR signaling pathway mediated by PDCD4. (A) Western blot analysis of the levels of p-AKT and p-mTOR in HEK293T cells overexpression with NPM1 and NPMc+. (B) Western blot analysis of the levels of p-AKT and p-mTOR in HEK293T cells overexpression with PDCD4. (C) As in A, but in PDCD4 knock down cells. (D) Western blotting detected the expression level of p-AKT and p-mTOR in HEK293T cells overexpression with GFP-NPMc+ alone or co-expression with NPMc+ and PDCD4.

Fig. S3

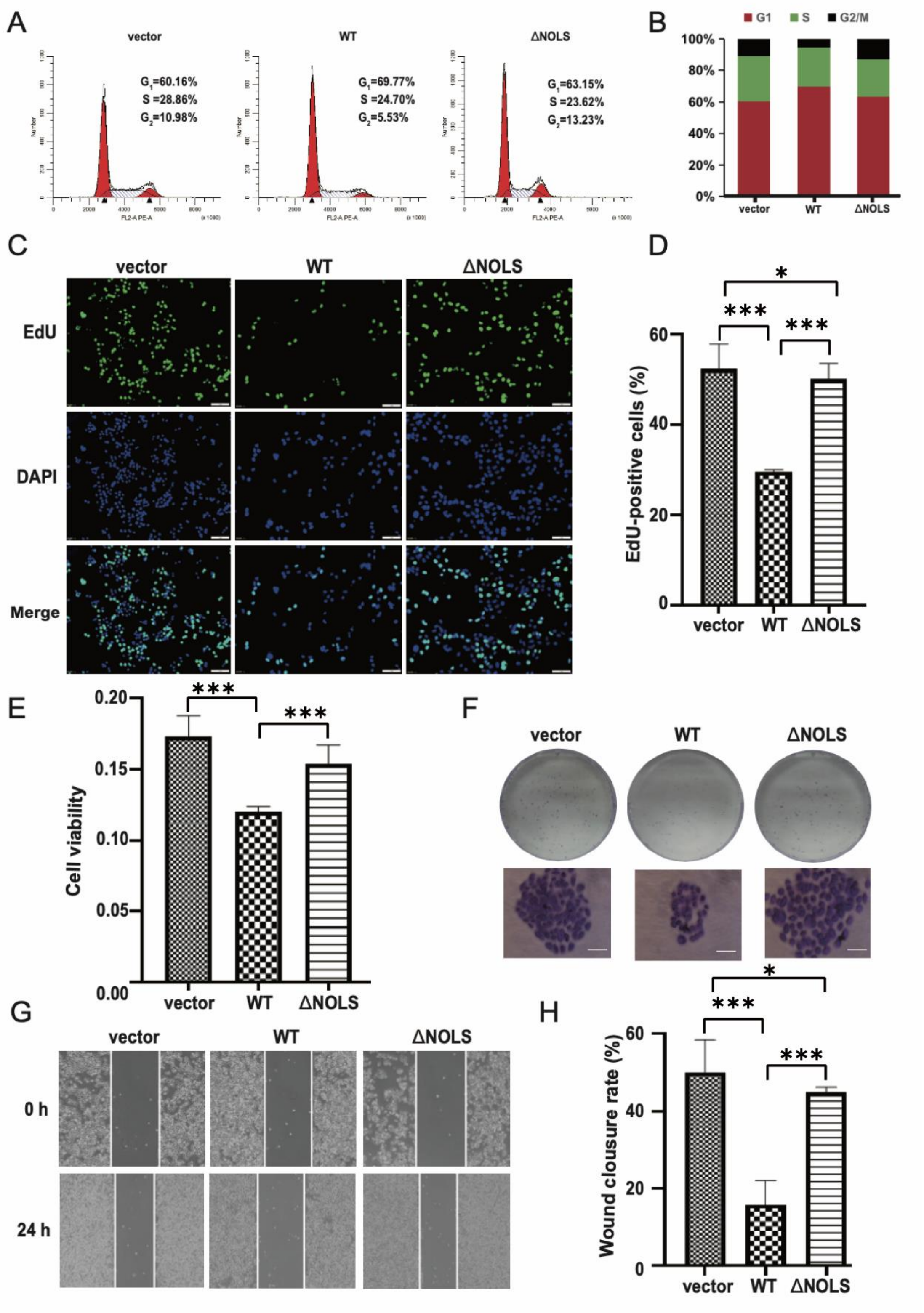

Fig. S3 Deletion of the NoLS sequence in the PDCD4 protein lost the tumor suppressive function. (A) Flow cytometric analysis of cell cycle distribution of HeLa cells overexpression with PDCD4 wildtype or PDCD4-ΔNoLS proteins. (B) Cell cycle distribution (G1, S, G2/M). PDCD4

effectively induced a G1 cell cycle arrest. However, no prominent change was observed in PDCD4-ΔNoLS overexpressing cells. (C) EdU staining for evaluation of the influences of PDCD4-ΔNoLS on the proliferation of HeLa cells. Dividing cells were labeled with EdU (green). All cells were counterstained with DAPI (blue). Scale bar: 50 μm. (D) Quantitative analyses of the percentages of EdU-positive cells. (E) MTT assay assessed the effect of PDCD4-ΔNoLS on the proliferation of HeLa cells. (F) Colony formation assay of HeLa cells overexpression with PDCD4 and PDCD4-ΔNoLS. Scale bar: 50 μm. (G) Scratch assay to evaluate migration ability of HeLa cells overexpression with PDCD4 and PDCD4-ΔNoLS. (H) Wound closure rates were expressed as percentages of the wound area closed at 24 h relative to the initial area at 0 h. All data are presented as mean ± SD of three independent experiments. p value is calculated using one-way ANOVA. $*p < 0.05$ and $***p < 0.001$.

Fig. S4

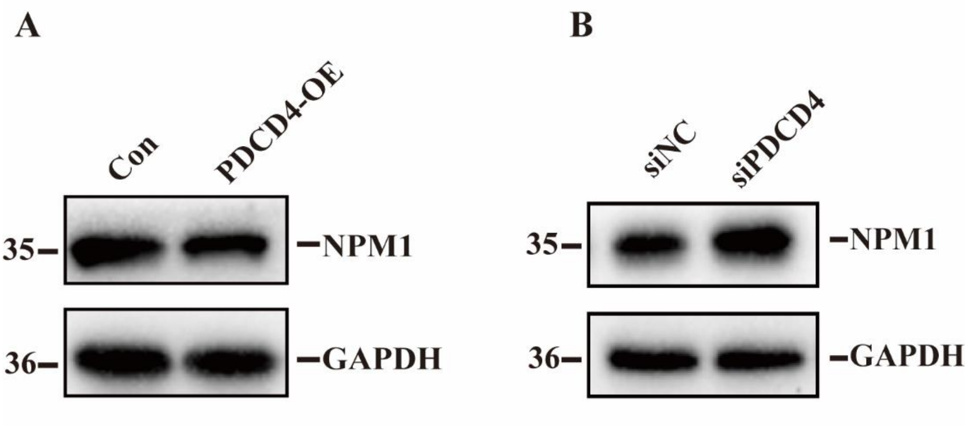

Fig. S4 PDCD4 regulates the expression of NPM1. (A) Western blot analysis of the levels of NPM1 in HEK293T cells overexpression with PDCD4. (B) As in A, but in PDCD4 knock down cells.

Data S1. Raw, uncropped images of the gels and immunoblot membranes.

Fig.1A

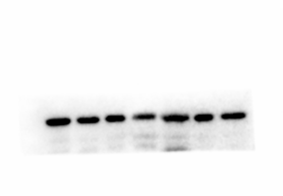 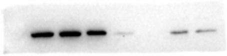 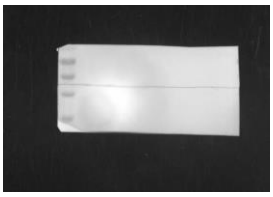

GAPDH PDCD4 Marker

Fig.1B

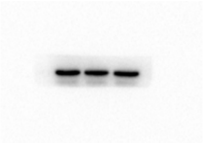 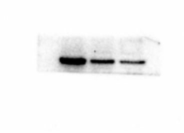 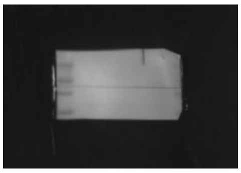

GAPDH PDCD4 Marker

Fig.1C

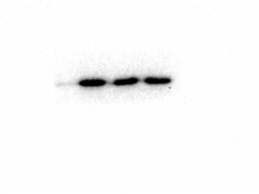 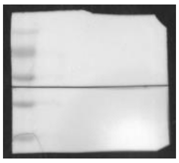

GAPDH PDCD4 Marker

Fig.2A **Fig.2B**

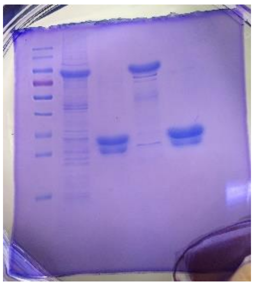

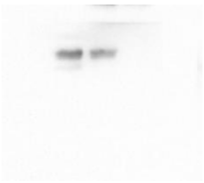

Fig.2C

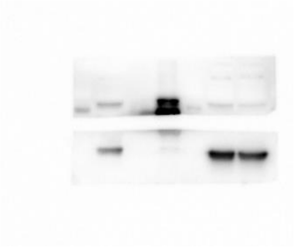

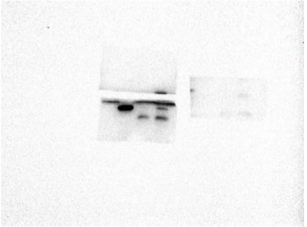

Fig.2D **Fig.2E** **Fig.2I**

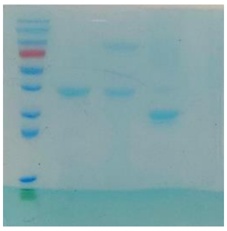

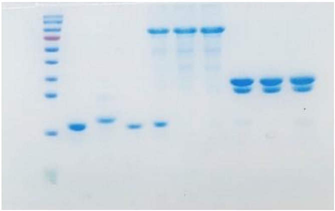

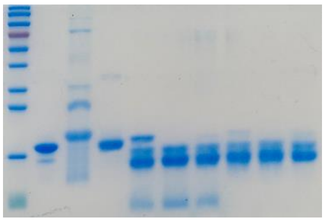

Fig.3D

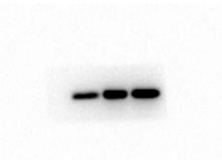

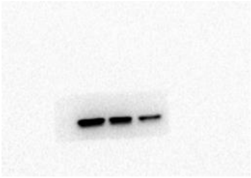

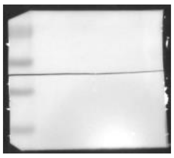

GAPDH PDCD4 Marker

Fig.3E

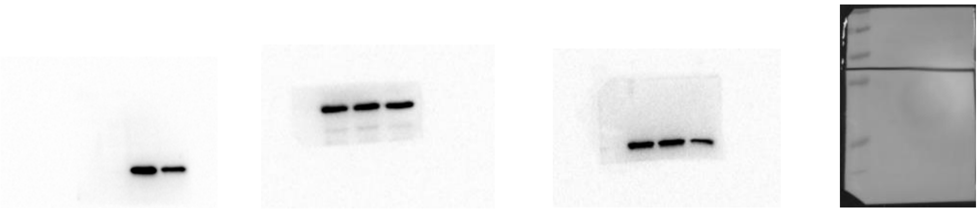

Flag-NPM1 GAPDH PDCD4 Marker

Fig.4A **Fig.4B**

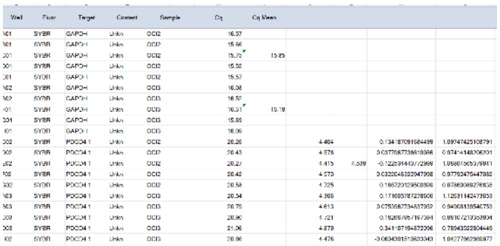

Well	Fluor	Target	Content	Sample	Cq	Cq Mean				
[illegible]01	SYBR	GAPDH	Unkn	OCI2	16.57					
[illegible]01	SYBR	GAPDH	Unkn	OCI2	15.66					
[illegible]01	SYBR	GAPDH	Unkn	OCI2	15.75	15.85				
[illegible]01	SYBR	GAPDH	Unkn	OCI2	15.52					
[illegible]01	SYBR	GAPDH	Unkn	OCI2	15.57					
[illegible]02	SYBR	GAPDH	Unkn	OCI3	16.08					
[illegible]02	SYBR	GAPDH	Unkn	OCI3	16.52					
[illegible]01	SYBR	GAPDH	Unkn	OCI3	16.31	16.18				
[illegible]01	SYBR	GAPDH	Unkn	OCI3	15.89					
[illegible]01	SYBR	GAPDH	Unkn	OCI3	16.06					
[illegible]02	SYBR	PDCD4 1	Unkn	OCI2	20.26		4.404		0.134187091684499	1.09747425108791
[illegible]02	SYBR	PDCD4 1	Unkn	OCI2	20.43		4.578		0.0377987738619086	0.97414148206201
[illegible]02	SYBR	PDCD4 1	Unkn	OCI2	20.27		4.415	4.539	-0.122594443772998	[illegible]
[illegible]02	SYBR	PDCD4 1	Unkn	OCI2	20.42		4.570		0.0322045322947998	0.97792475447082
[illegible]02	SYBR	PDCD4 1	Unkn	OCI2	20.58		4.725		0.186720128500898	0.87880089278835
[illegible]03	SYBR	PDCD4 1	Unkn	OCI3	20.54		4.365		0.171605787278506	1.12631142473653
[illegible]03	SYBR	PDCD4 1	Unkn	OCI3	20.79		4.613		0.0753987734837952	0.94908139540753
[illegible]03	SYBR	PDCD4 1	Unkn	OCI3	20.90		4.721		[illegible]	[illegible]
[illegible]03	SYBR	PDCD4 1	Unkn	OCI3	21.06		4.879		0.341107194672096	0.78943522804449
[illegible]02	SYBR	PDCD4 1	Unkn	OCI3	20.66		4.476		[illegible]	1.04277862868977

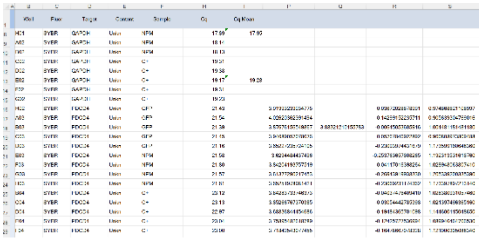

	Well	Fluor	Target	Content	Sample	Cq	Cq Mean				
8	H01	SYBR	GAPDH	Unkn	NPM	17.89	17.95				
9	A02	SYBR	GAPDH	Unkn	NPM	18.14					
10	[illegible]	SYBR	GAPDH	Unkn	NPM	[illegible]					
11	C02	SYBR	GAPDH	Unkn	C+	19.31					
12	D02	SYBR	GAPDH	Unkn	C+	19.38					
13	E02	SYBR	GAPDH	Unkn	C+	19.17	19.28				
14	F02	SYBR	GAPDH	Unkn	C+	19.31					
15	G02	SYBR	GAPDH	Unkn	C+	[illegible]					
16	H02	SYBR	PDCD4	Unkn	GFP	21.43		[illegible]		[illegible]	[illegible]
17	A03	SYBR	PDCD4	Unkn	GFP	21.54		4.02620982391494		[illegible]	[illegible]
18	B03	SYBR	PDCD4	Unkn	GFP	21.38		[illegible]	[illegible]	[illegible]	[illegible]
19	C03	SYBR	PDCD4	Unkn	GFP	21.45		[illegible]		[illegible]	[illegible]
20	D03	SYBR	PDCD4	Unkn	GFP	21.16		[illegible]		[illegible]	[illegible]
21	E03	SYBR	PDCD4	Unkn	NPM	21.58		[illegible]		[illegible]	[illegible]
22	F03	SYBR	PDCD4	Unkn	NPM	21.80		[illegible]		[illegible]	[illegible]
23	G03	SYBR	PDCD4	Unkn	NPM	21.57		[illegible]		[illegible]	[illegible]
24	H03	SYBR	PDCD4	Unkn	NPM	21.81		[illegible]		[illegible]	[illegible]
25	B04	SYBR	PDCD4	Unkn	C+	23.12		[illegible]		[illegible]	[illegible]
26	C04	SYBR	PDCD4	Unkn	C+	23.13		[illegible]		[illegible]	[illegible]
27	D04	SYBR	PDCD4	Unkn	C+	22.97		[illegible]		[illegible]	[illegible]
28	F04	SYBR	PDCD4	Unkn	C+	[illegible]		[illegible]		[illegible]	[illegible]
29	[illegible]	SYBR	PDCD4	Unkn	C+	[illegible]		[illegible]		[illegible]	[illegible]

Fig.4C

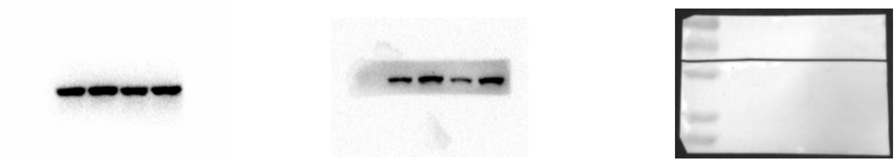

GAPDH PDCD4 Marker

Fig.4D

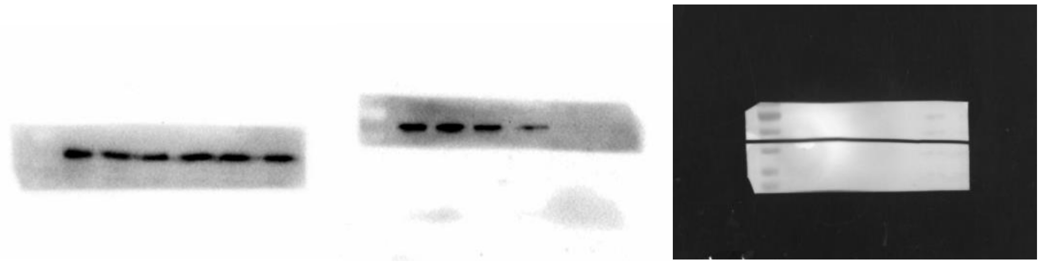

GAPDH PDCD4 Marker

Fig.4E

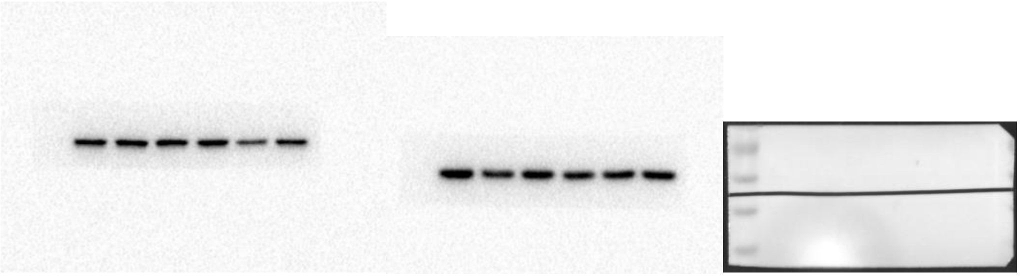

GAPDH PDCD4 Marker

Fig.4F

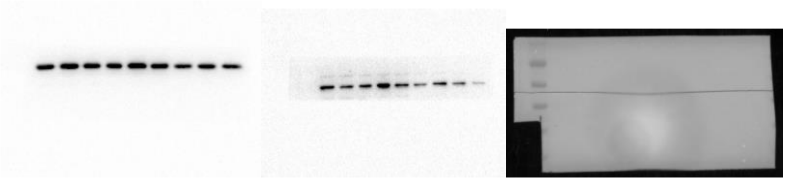

GAPDH PDCD4 Marker

Fig.4G

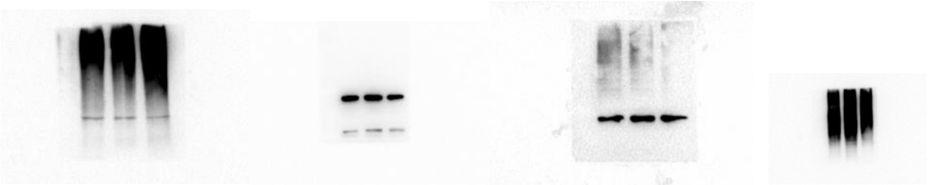

IP： strep WB: HA, strep Input WB: strep, HA

Fig.5A

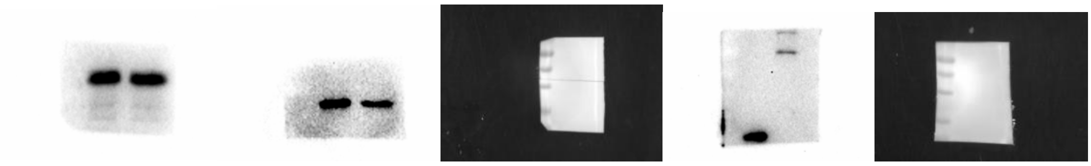

GAPDH PDCD4 Marker GFP-NPMc+ Marker

Fig.5F

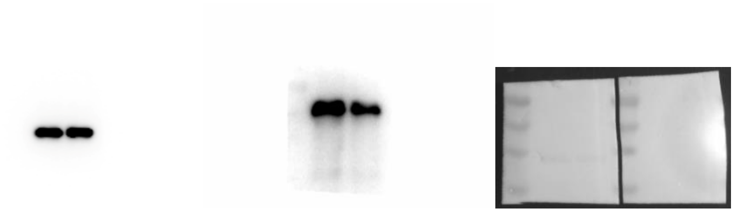

Flag PDCD4 Marker

Fig.5G

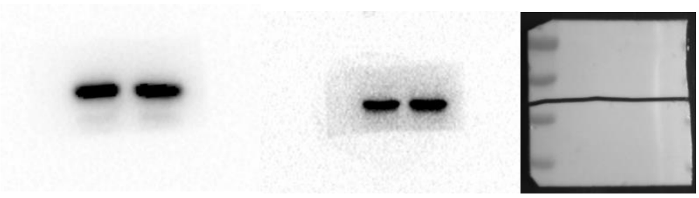

GAPDH PDCD4 Marker

Fig.6A **Fig.6B**

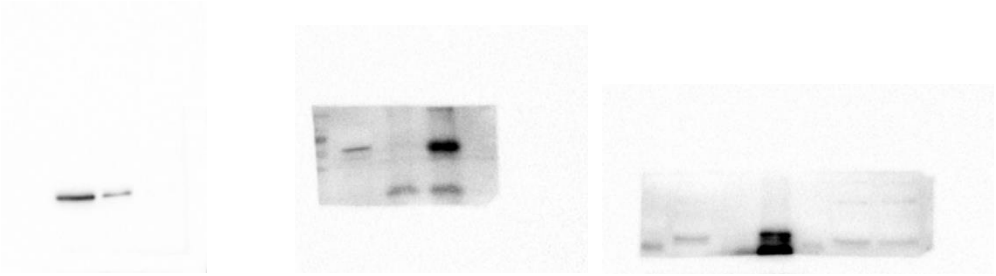

Fig.6D **Fig.6F**

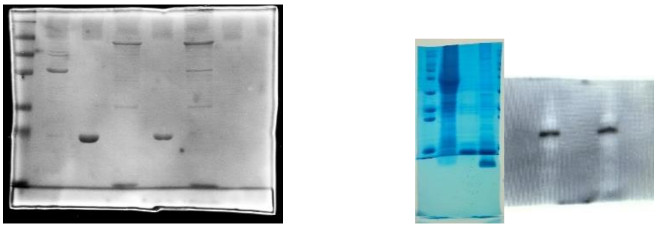

Fig.6G

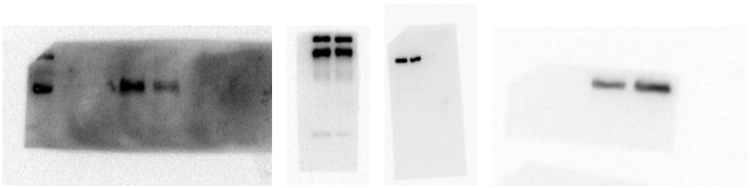

Fig.7E

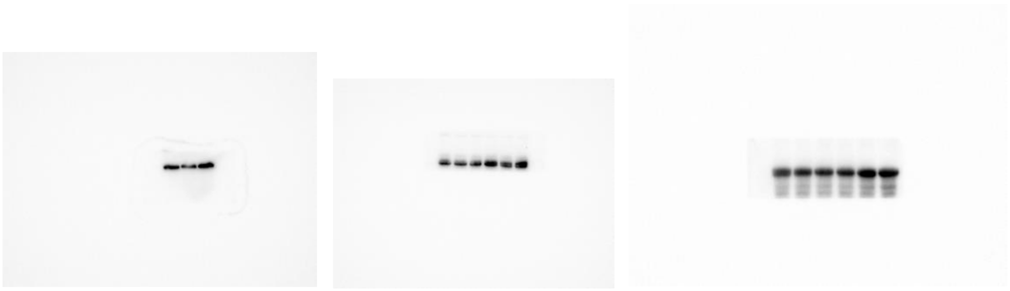

Hox1 Meis1 GAPDH

Fig.7F

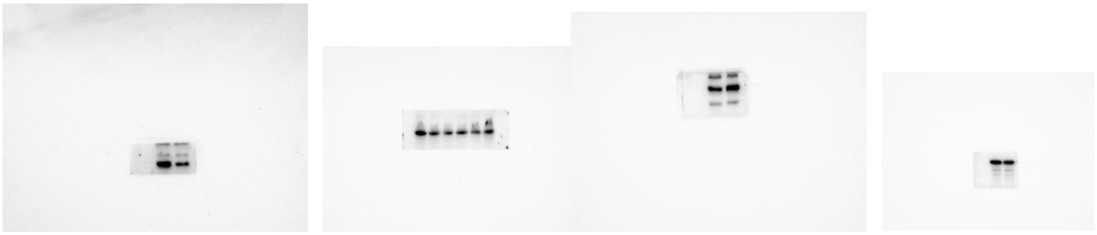

Meis1 Hox1 PDCD4 GAPDH

Fig.7G

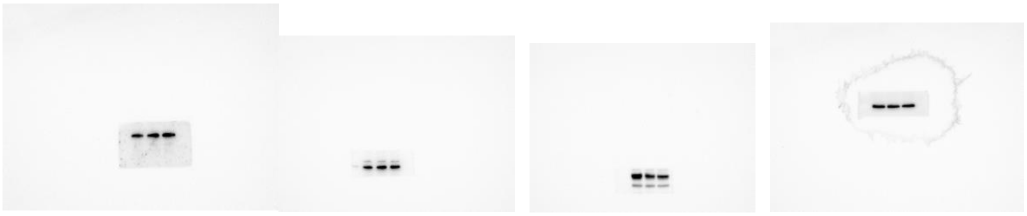

Hox1 Meis1 PDCD4 GAPDH

Fig.7H

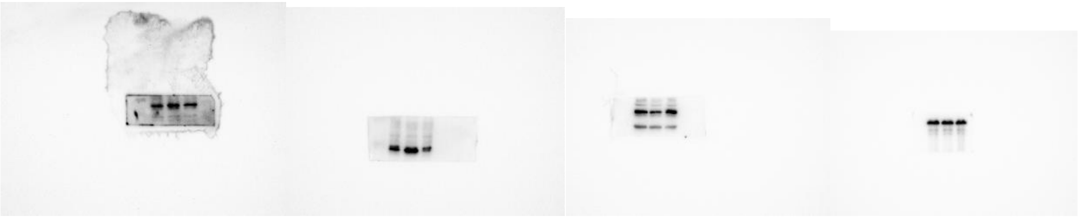

Hox1 Meis1 PDCD4 GAPDH

Fig.7I

Well	Fluor	Target	Content	Sample	Cq	Cq Mean	Cq Std. Dev
B04	SYBR	GAPDH	Unkn	NC	15.19	15.19	0.000
B05	SYBR	GAPDH	Unkn	P1	16.17	16.17	0.000
B06	SYBR	GAPDH	Unkn	P2	16.45	16.45	0.000
B07	SYBR	MESI	Unkn	NC	25.31	25.31	0.000
B08	SYBR	MESI	Unkn	P1	26.10	26.10	0.000
B09	SYBR	MESI	Unkn	P2	27.00	27.00	0.000
C04	SYBR	GAPDH	Unkn	NC	15.16	15.16	0.000
C05	SYBR	GAPDH	Unkn	P1	16.10	16.10	0.000
C06	SYBR	GAPDH	Unkn	P2	16.39	16.39	0.000
C07	SYBR	MESI	Unkn	NC	25.26	25.26	0.000
C08	SYBR	MESI	Unkn	P1	26.11	26.11	0.000
C09	SYBR	MESI	Unkn	P2	27.04	27.04	0.000
D04	SYBR	GAPDH	Unkn	NC	15.39	15.39	0.000
D05	SYBR	GAPDH	Unkn	P1	16.14	16.14	0.000
D06	SYBR	GAPDH	Unkn	P2	16.41	16.41	0.000
D07	SYBR	MESI	Unkn	NC	25.30	25.30	0.000
D08	SYBR	MESI	Unkn	P1	25.97	25.97	0.000
D09	SYBR	MESI	Unkn	P2	27.07	27.07	0.000
E04	SYBR	NPM	Unkn	NC	16.21	16.21	0.000
E05	SYBR	NPM	Unkn	P1	17.31	17.31	0.000
E06	SYBR	NPM	Unkn	P2	17.85	17.85	0.000
E07	SYBR	HOX	Unkn	NC	27.16	27.16	0.000
E08	SYBR	HOX	Unkn	P1	27.42	27.42	0.000
E09	SYBR	HOX	Unkn	P2	28.04	28.04	0.000
F04	SYBR	NPM	Unkn	NC	16.54	16.54	0.000
F05	SYBR	NPM	Unkn	P1	17.24	17.24	0.000
F06	SYBR	NPM	Unkn	P2	17.86	17.86	0.000
F07	SYBR	HOX	Unkn	NC	26.90	26.90	0.000
F08	SYBR	HOX	Unkn	P1	27.52	27.52	0.000
F09	SYBR	HOX	Unkn	P2	28.02	28.02	0.000
G04	SYBR	NPM	Unkn	NC	16.24	16.24	0.000
G05	SYBR	NPM	Unkn	P1	17.17	17.17	0.000
G06	SYBR	NPM	Unkn	P2	17.51	17.51	0.000
G07	SYBR	HOX	Unkn	NC	26.89	26.89	0.000
G08	SYBR	HOX	Unkn	P1	27.44	27.44	0.000
G09	SYBR	HOX	Unkn	P2	28.21	28.21	0.000
